# Analyzing Secondary Structure Patterns in DNA Aptamers Identified via CompELS

**DOI:** 10.3390/molecules24081572

**Published:** 2019-04-21

**Authors:** Richard Sullivan, Mary Catherine Adams, Rajesh R. Naik, Valeria T. Milam

**Affiliations:** 1 School of Materials Science and Engineering, Georgia Institute of Technology, 771 Ferst Dr. NW, Atlanta, GA 30332-0245, USA; rsulliv@gatech.edu (R.S.); madams63@gatech.edu (M.C.A.); 2 711 Human Performance Wing, Air Force Research Laboratory, Wright Patterson AFB, OH 45433, USA; rajesh.naik@us.af.mil; 3 Wallace H. Coulter, Department of Biomedical Engineering, Georgia Institute of Technology, 313 Ferst Dr., Atlanta, GA 30332, USA; 4 Petit Institute for Bioengineering and Bioscience, Georgia Institute of Technology, 315 Ferst Dr., Atlanta, GA 30332-0363, USA

**Keywords:** aptamers, single-stranded DNA, nucleic acid ligands, secondary structure

## Abstract

In contrast to sophisticated high-throughput sequencing tools for genomic DNA, analytical tools for comparing secondary structure features between multiple single-stranded DNA sequences are less developed. For single-stranded nucleic acid ligands called aptamers, secondary structure is widely thought to play a pivotal role in driving recognition-based binding activity between an aptamer sequence and its specific target. Here, we employ a competition-based aptamer screening platform called CompELS to identify DNA aptamers for a colloidal target. We then analyze predicted secondary structures of the aptamers and a large population of random sequences to identify sequence features and patterns. Our secondary structure analysis identifies patterns ranging from position-dependent score matrixes of individual structural elements to position-independent consensus domains resulting from global alignment.

## 1. Introduction

Explosive progress in high-throughput DNA sequencing has driven advances in analytical tools to identify base consensus motifs among subgroups of DNA sequences [1,2] as well as to identify evolutionary connections between larger groups of sequences using global alignment [3,4,5] approaches. First reported in 1994, ClustalW [3] endures as one of the most cited [6] multiple sequence alignment tools. Over the last 25 years, updated algorithms continue to refine the alignment process [7,8] to accommodate analysis demands for large datasets of biologically relevant sequences to identify any shared genomic aspects. These sequence analysis tools can also be employed to identify patterns among nongenomic yet functional oligonucleotides called aptamers. Aptamers are single-stranded oligonucleotide sequences that bind to a particular target (small molecules, proteins, etc.,) with high affinity and specificity with applications ranging from sensors [9,10,11,12] to therapeutics [13,14,15]. Traditionally, aptamers are identified via an evolutionary selection process first reported separately by three groups in 1990 [16,17,18]. The term “systematic evolution of ligands by exponential enrichment”, or SELEX, was coined by Tuerk and Gold to describe their screening process [16]. In the decades that followed, numerous groups have reported new aptamer sequences identified via SELEX for a variety of non-nucleotide targets. Though aptamers are nongenomic sequences, tools built for genomic sequence analysis can still be useful, as demonstrated in studies with RNA aptamers [19,20,21,22,23].

In contrast to advances in sequence analysis tools, progress lags in predictive secondary structure tools for self-hybridized, single-stranded DNA (ssDNA). Published literature (e.g., Dunaway et al. [24]) typically employs modeling tools such as mfold [25] to predict and illustrate the overall 2D self-hybridized conformations of individual aptamer sequences. Relatively few studies [13,26,27], however, identify specific patterns in individual secondary structure features or elements among multiple self-hybridized DNA structures. Similarly, experimental work to verify secondary structure predictions from these models is generally lacking for single-stranded DNA. Exceptions to this information gap tend to reside with historically popular DNA aptamers such as Bock’s thrombin aptamer [28,29,30] or with studies focused on identifying potentially shared binding motifs [26,27] among different DNA aptamer sequences for the same target. Such structural information can allow guided DNA aptamer truncation efforts [27,31,32] or strategic extension of hybridized segments to stabilize self-hybridized structures [27]. In the absence of such information, one must otherwise undertake arduous trial-and-error experiments to unmask the binding motifs in shortened, often higher affinity [31,33] DNA aptamer segments.

In contrast to DNA aptamers, structural analysis has informed design strategies for RNA-based aptamers using comprehensive three-dimensional structural databases both for RNA alone [34,35] and also RNA–protein complexes [36,37,38]. Moreover, studies by Hoinka et al. [19] on RNA aptamer systems expand their analysis by considering both primary structure and secondary structure. Using this approach, they can identify base consensus motifs among unhybridized bases present in the loop segment of a self-hybridized RNA hairpin. Despite the abundance of aptamer SELEX screening studies, the paucity of structure-function information in DNA aptamer systems can be attributed, at least in part, to two interconnected challenges: (1) The nontrivial nature of these characterization pursuits (as demonstrated with multidimensional NMR spectroscopy studies of RNA aptamers [39,40]) and (2) the lack of three-dimensional databases and predictive tools for self-hybridized DNA sequences.

To begin closing this analytical gap in understanding the specific role that secondary structure may play in DNA aptamer-target binding, Tapp et al. [41] defined a classification scheme to identify shared features and patterns in predicted minimum or lowest free energy self-hybridized structures among DNA aptamer sequences. Importantly, in contrast to employing traditional SELEX screening, Tapp et al. [41] developed a non-evolutionary aptamer screening platform called competition-enhanced ligand screening or CompELS in order to identify aptamer candidates for subsequent analysis. Here, the current work expands on Tapp’s secondary structure analysis by adapting existing motif identification and multiple protein sequence alignment tools in order to score the occurrence of both dominant and suboptimal secondary structure elements and to identify shared domains where particular secondary structure features predominate. Notably, while analytical tools for aligning pairs [42,43] as well as groups [44,45,46] of RNA secondary structure features and even suboptimal self-hybridized structures [47,48] are available, these tools generally presume an evolutionary connection between biologically relevant RNA sequences [49]. Additionally, some RNA alignment programs take into account secondary structure features such as pseudoknots [50] more widely reported in RNA sequences. In light of the nonevolutionary nature of the CompELS screening process used in the current work as well as the structural features better catalogued in previously mentioned RNA databases [34,35,36,37], the analytical approach reported here strives to avoid introducing potential artifacts while still leveraging the capabilities of existing well-cited analytical tools. Here, position-specific score matrices (PSSM) [51,52] and ClustalW [3] are the analytical tools adapted to define if any correlations or patterns in predicted self-hybridized structures exist between DNA aptamers identified via CompELS for gold nanorod (AuNR) targets.

## 2. Results and Discussion

### 2.1. DNA Aptamers for AuNR Identified Using CompELS Screening Platform

**Scheme 1:**
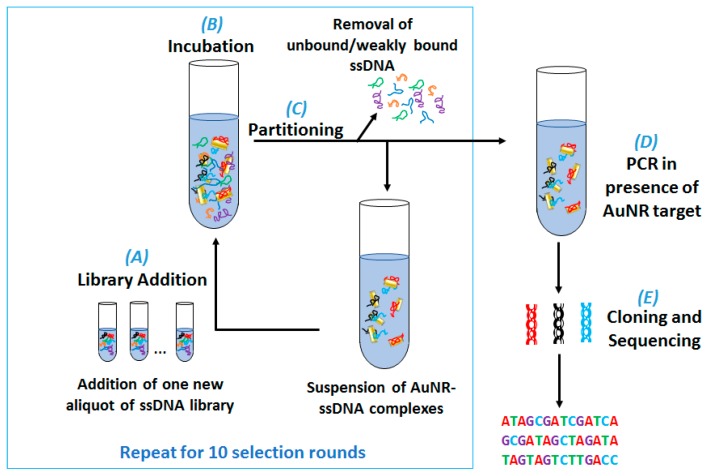
Competition-enhanced ligand screening (CompELS) aptamer selection for DNA aptamers against gold nanorods (AuNR) that entails (**A**) addition of one single-stranded DNA (ssDNA) library aliquot to (**B**) AuNR followed by (**C**) removal of unbound, weakly bound, or displaced DNA sequences. Following the completion of 10 rounds of CompELS, (**D**) PCR is carried out in the presence of AuNR and, finally, (**E**) cloning and sequencing of aptamer candidates is undertaken.

AuNR [53,54,55,56,57,58,59] possess an array of advantageous properties that allow for diverse bio-based applications ranging from biosensing to therapeutics. Tunable radiative and nonradiative properties in AuNR suspensions are attributed to both their anisotropic shape possibilities and their noble metal composition. In the current work, AuNR serve as the colloidal targets for aptamer screening. UV-vis spectroscopy of AuNR targets is provided in Figure S1 in supplementary materials. DNA aptamer selection was performed in three separate CompELS screening sessions illustrated in Scheme 1 using ssDNA random libraries with an equidistribution of A, T, C, and G bases for the first two screenings (resulting in aptamer sequence sets 1XX and 2XX) and using a ssDNA A-rich random library for the third screening (resulting in aptamer sequence set 4XX).

The concentration of ssDNA (in each library aliquot) was chosen to allow for excess ssDNA to surround suspended AuNR (~10^10^ particles). Based on prior TEM characterization [60], seeded AuNR synthesis yielded ~21 nm diameter rods with an average length of ~57 nm (aspect ratio ~2.7), which gives an estimated surface area of ~4450 nm^2^ per AuNR available for DNA binding events. Using a freely jointed chain model for a 69 base-long DNA strand with an overall random coil conformation, the radius of gyration, *R_g_*, is estimated to be ~3.32 nm for each ssDNA strand from the library. Based on these estimates, the number of ssDNA strands used for panning in each CompELS round was ~100X excess of that required to form a monolayer of close-packed ssDNA random coils “spheres” on AuNR in solution. After each incubation with a library aliquot, the suspension was centrifuged, DNA strands remaining in the supernatant were removed, and a new aliquot of ssDNA library was introduced to the AuNR for the next CompELS round. Importantly, no additional stringent wash steps were undertaken to intentionally dissociate and then amplify the population of DNA strands remaining from prior CompELS rounds. This screening approach differs from SELEX, in which bound sequences are eluted, recovered, and then amplified via PCR during each screening round to enrich the subsequent candidate pool with prior adsorbate species. After 10 selection rounds, six washes of AuNR suspensions with the aptamer binding buffer (ABB) were completed to remove any remaining weakly bound DNA adsorbate species. Lastly, PCR was performed in the presence of the AuNR for recovery, vector insertion, cloning, and sequencing of bound DNA strands. A total of 42 DNA aptamer sequences from the three separate screenings are listed in Table S1 in supplementary materials and collectively analyzed. Multiple sequence alignment results from T Coffee analysis on the central 40 base-long segments in Figure S2 in supplementary materials indicate up to 69% consensus in select individual base identities. Separate MEME (Multiple EM (Expectation Maximization) for Motif Elicitation) analysis (not shown), however, does not reveal any statistically significant consensus motifs across two or more neighboring bases among the 42 aptamer sequences.

### 2.2. Assigning Subgroups of Aptamer sequeNces to a Secondary Structure Family (SSF)

**Scheme 2:**
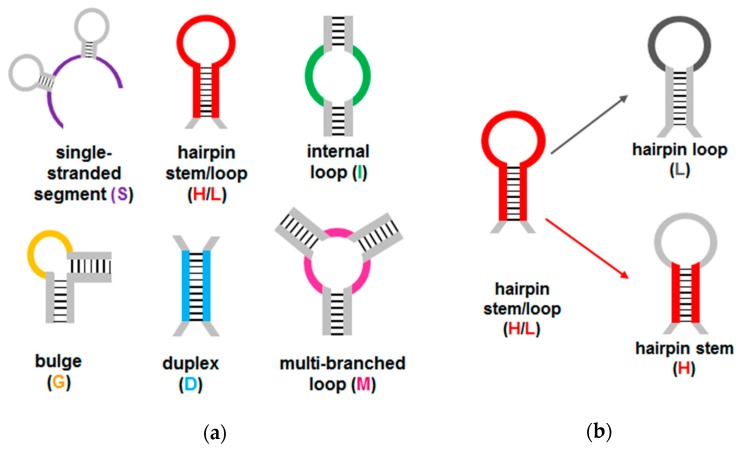
Color-coded illustrations and abbreviations of (**a**) six predicted secondary structure elements (SSE) namely, single-stranded segment (purple), hairpin stem/loop (red), internal loop (green), bulge (yellow), duplex (blue), and multi-branched loop (pink) used to identify each secondary structure family (SSF) among AuNR aptamer candidates. (**b**) Each hairpin is further broken down into a hairpin loop (gray) and hairpin stem (red) for multiple secondary structure string alignment (MSS$A) analysis.

**Figure 1:**
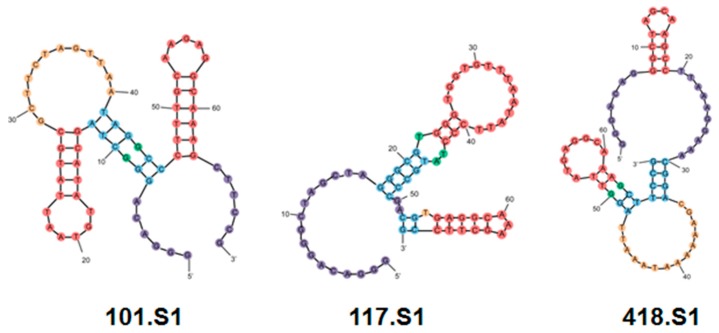
Schematics of three members of self-hybridized AuNR aptamer sequences, namely, 101.S1, 117.S1, and 418.S1 of SSF2. As shown in Table 1, each member of SSF2 possesses two single-stranded segments (purple); two hairpin stem/loops (red); one internal loop (green); one bulge (yellow); two duplexes (blue); and zero multi-branched loops (dark pink).

Based on a prior classification scheme reported by Tapp et al. [41], each predicted self-hybridized structure for an aptamer sequence was broken down into different secondary structure elements (SSE) illustrated in Scheme 2. Two or more aptamer sequences were then assigned to the same secondary structure family (SSF) if they shared the exact same numbers of each unique SSE. The dominant and/or suboptimal secondary structures of a given aptamer sequence listed Table S2 in supplementary materials can be members of an SSF. Finally, the base length, sequence content, and base position of each SSE were not taken into account in assigning SSF. Based on these criteria described above, 11 groups of SSF listed in Table 1 were identified among the 42 AuNR aptamer sequences. Figure 1 shows color-coded schematics of the predicted secondary structures of three members of one family, namely SSF2, that exhibit all SSE except multi-branched loops. Schematics of members of all 11 SSF are shown in Figure S3 in supplementary materials. As detailed in the Experimental Methods section, the one-letter abbreviation for each SSE shown in Scheme 2 also corresponds to the one-letter substitution used to create secondary structure strings (SS$) for each predicted dominant and, if relevant, suboptimal structure(s) for each aptamer sequence discussed next.

### 2.3. Mapping Secondary Structure Elements (SSE) at Each Base Position

**Figure 2:**
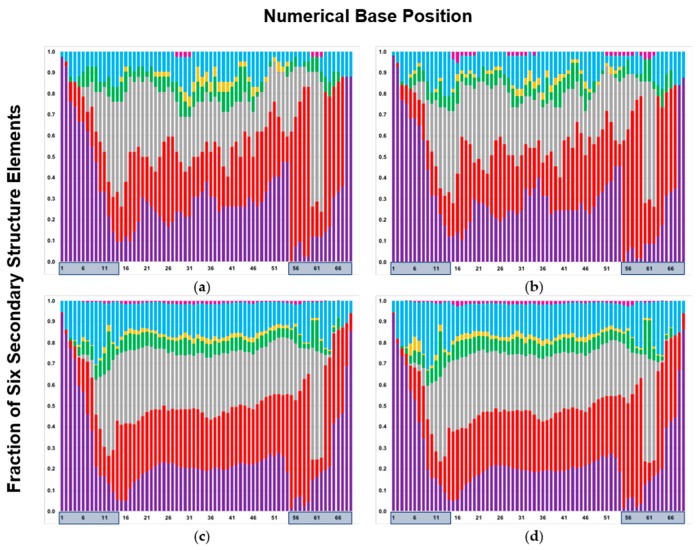
Distribution of secondary structure elements (SSE) as a function of numerical base position for self-hybridized (**a**) dominant-only structures of the aptamers, (**b**) dominant + suboptimal structures of the aptamers, (**c**) dominant-only structures of a random sequence population, and (**d**) dominant + suboptimal structures of a random sequence population. Each bar is color-coded to indicate the relative fraction of multi-branched loops (dark pink); duplexes (blue); bulges (yellow); internal loops (green); hairpin loops (gray); hairpin stems (red); and single-stranded segments (purple) at a given base position. Numerical base positions corresponding to the two fixed base segments are highlighted in gray on each x-axis.

In contrast to assigning 36 (out of 42 total) dominant and 10 (out of 14 total) suboptimal secondary structures to 11 distinct SSF groupings, the next analysis involves identifying global patterns among all predicted secondary structures of the aptamer sequences. To undertake this global alignment process (discussed in the next section), first the frequency for each SSE occurrence at each base position is mapped for dominant-only self-hybridized structures in Figure 2a and dominant + suboptimal self-hybridized structures in Figure 2b for all aptamers. To create a background for analytical purposes, dominant-only self-hybridized structures in Figure 2c and dominant + suboptimal self-hybridized structures in Figure 2d are also mapped for a large (10^3^) random sequence population. Notably, the two fixed base segments (flanking the central 40 base-long variable segment) are identical for both aptamers and the random sequence population. The percentage differences in SSE frequency (ΔSSE(%)) between the dominant-only and the dominant + suboptimal structures for aptamers and the random sequence population are plotted in Figures S4 and S5, respectively, in supplementary materials. From collective examination of all four cases shown in Figure 2a–d, Figures S4 and S5, some shared and unshared trends in the distribution of SSE can be ascertained. Shared trends in the base position-dependent distribution of SSE include the following: (1) Single-stranded segments occur at nearly every base position and especially predominate near the 5’ end; and (2) hairpin stems and hairpin loops are the next most frequent SSE with prevalence near the 3’ end. As a more specific example of this shared pattern, a hairpin loop structure predominates at base positions 60–62 with the flanking hairpin stem at base positions 55–59 and 63–67. As exhibited by several self-hybridized aptamers (e.g., 111.S3, 120.S1, 214.S1, 412.S2 in SSF3 in Figure S3 in supplementary materials) this hairpin at bases 55–67 includes a three adenine-long loop. Many of these shared features near the 5′ and 3′ ends are likely due to their identical primary structure; however, base pairings also frequently occur between the fixed base segment (bases 1–14 and 55–69) and central variable segment (bases 15–54) to form, for example, duplexes (e.g., bases 8 and 45 in 101.S1 shown in Figure 1) or hairpin stems (e.g., bases 8 and 19 in 418.S1 in Figure 1).

While there are numerous other examples of shared trends, there are also distinctions in SSE distributions between the aptamers in Figure 2a,b and the random sequences in Figure 2c,d. These distinctions occur in both in the fixed base segments as well as in the central variable base segments. General differences are as follows: (1) The inclusion of suboptimal structures generally results in more frequent occurrence of multi-branched loops and bulges in aptamers while these same SSE occur frequently in both the dominant-only as well as dominant + suboptimal structures for the random sequence population; and (2) a richer variety of SSE at nearly all base positions occurs in the random sequence population than in the aptamer population. As just one example of this second generality, base 21 is comprised of single-stranded segments, hairpin stems, hairpin loops, internal loops, and duplexes among dominant as well as dominant + suboptimal aptamer structures, whereas the random sequence population additionally includes a small fraction of both multi-branched loops and bulges at this same base position for both dominant-only and dominant + suboptimal structures. More specific examples of quantitative differences in the frequency of each SSE at several base positions for aptamers and the random sequence population, particularly hairpin stems and single-stranded segments, are evident in bar graphs shown in Figures S4 and S5 in supplementary materials.

### 2.4. Using Random Sequence Populations to Generate Position-Specific Score Matrices (PSSM) of SSE in Aptamers

**Figure 3:**
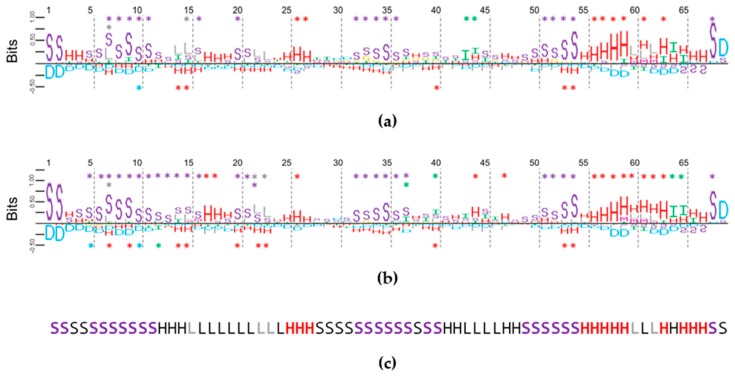
Position specific score matrix (PSSM) of SSE for (**a**) dominant-only and (**b**) dominant + suboptimal structures of aptamers using the random sequence population as the background and α = 1 in the additive smoothing function. A higher bit score either positive (i.e., stronger presence) or negative (i.e., stronger absence) for one or more SSE at a particular numerical base position corresponds to a greater height of the one-letter symbol for a given SSE. Numerical base positions with significant differences in a given SSE determined separately using a one-tailed two proportion Z-test (shown in Figures S4 and S5, respectively, in supplementary materials) are marked with an asterisk. (**c**) secondary structure strings (SS$) for aptamer 201.S1 with highest scoring match to both PSSM shown above. Individual position-specific matches in SSE between PSSM and 201.S1 SS$ are color-matched while nonmatching SSE are shown in black text in (**c**).

After examining strictly numerical differences in SSE occurrences at each base position in the aptamer and random sequence populations, a more weighted approach was undertaken to compute a position specific score matrix (PSSM) as detailed in Experimental Methods. The resulting PSSM for aptamers are shown in Figure 3a for dominant-only structures, and in Figure 3b for dominant + suboptimal structures.

The PSSM for dominant-only secondary structures in Figure 3a shows close agreement overall in trends to ΔSSE shown in Figure S4 in supplementary materials. It should be noted that there are fewer potential SSE possibilities at and near the 5’ and 3’ ends (e.g., a bulge cannot occur at base 1 or 69); however, each category of SSE shown in Scheme 2 appears in both PSSM plots. Thus, any differences in SSE at these ends between the aptamer and the random sequence background become more heavily weighted resulting in higher bit scores for the presence of single-stranded segments (indicated above each x-axis) and absence of duplexes (indicated below each x-axis) at the 5’ end while both SSE are present near and at the 3’ end. At several positions, the PSSM reveals a weighted prominence of an element (e.g., internal loop at position 64 in Figure 3a) that may be overlooked due to its modest ΔSSE value (e.g., see ΔSSE value for internal loops at position 64 in Figure S4 in supplementary materials).

For PSSM that accounts for dominant + suboptimal structures in Figure 3b, there is also overall agreement with its related ΔSSE analysis shown in Figure S5 in supplementary materials. Figure 3a,b show single-stranded segments and hairpin stems as the more prominent elements at several positions, with other elements such as hairpin loops and internal loops only occasionally prominent. Overall, strong overlap in SSE identities occur in many numerical base positions in Figure 3a,b; however, occasional distinctions in SSE identities occur (e.g., internal loops at positions 64–65 in dominant-only in Figure 3a extend further to position 66 in dominant + suboptimal in Figure 3b). Finally, Figure 3c shows the SS$ for aptamer 201.S1 that has the closest match to both PSSM in Figure 3a,b.

### 2.5. Multiple Secondary Structure String Alignment (MSS$A) in Aptamers and Random Sequences

**Figure 4:**
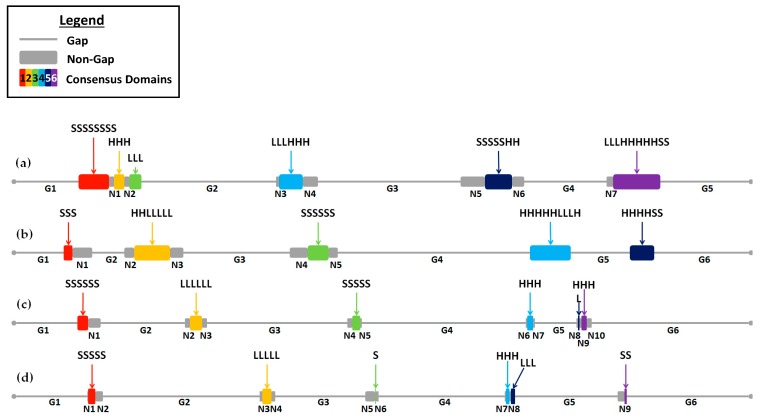
Linear representation of MSS$A that includes all gaps (G1, G2, etc.); non-gaps (N1, N2, etc.) and SS$ defining a given secondary structure consensus domain based on all (**a**) dominant-only structures for AuNR aptamers; (**b**) dominant + suboptimal structures for AuNR aptamers; (**c**) dominant-only structures for a random sequence population background; and (**d**) dominant + suboptimal structures for a random sequence population.

Finally, ClustalW was used to generate multiple sequence alignments of the four sets of SS$. As detailed in the Experimental Methods sections, once aligned, consensus domains comprised of a particular SS$ (present in ≥50% of the aligned secondary structures) are then represented using two-dimensional heat maps as shown in Figure S6a,b in supplementary materials for the aptamer dominant-only SS$ and the aptamer dominant + suboptimal SS$. To facilitate comparisons between the four separate alignment sets, namely, (a) dominant-only SS$ for aptamers; (b) dominant + suboptimal SS$ for aptamers; (c) dominant-only SS$ for random sequences; and (d) dominant + suboptimal SS$ for random sequences, collective information from each two-dimensional heat map is projected onto a line as shown in Figure 4a–d. In addition to showing all secondary structure consensus domains comprised of particular SS$ identities, these 1D representations also show where inserted gaps prevail (in ≥50% of aligned SS$) and non-gaps (where neither a gap nor a particular SSE or SS$ prevails) occur. In order to subsequently assess the base positions of these secondary structure consensus domains, the gaps are then removed for all four cases and then replotted in Figure S7a–d in the supplementary materials. The relative ratio of inclusion of each consensus domain as a function of base position is plotted separately in Figure S8a–d in the supplementary materials. Additional numerical data for each consensus domain are provided in Tables S3 and S4 in the supplementary materials. Collective observations stemming from MSS$A data in these multiple figures and tables are highlighted next.

In Figure 4a, the MSS$A for the dominant-only SS$ of AuNR aptamers identifies six consensus domains with the average number of consensus domains equal to 5.26 ± 0.99 per sequence. Overall, secondary structure consensus domains occupy an average of 37.9% of a given aptamer SS$ with the remainder comprised of non-gaps and gaps. Non-gaps are often next to or sandwiched between consensus domains, while gaps are often found between non-gaps. In the dominant-only SS$ for the random sequence population in Figure 4c, six consensus domains exist in the MSS$A with an average number of consensus domains equal to 4.70 ± 1.05 per sequence. On average, these consensus domains occupy only 23.7% of a given random sequence SS$ and may point to a larger degree of SS$ disorder in the random sequence population compared to the aptamers. Unlike the PSSM shown in Figure 3a,b, in which each SSE is present at one or more numerical base positions, the SS$ of each consensus domain in all four sets of MSS$A in Figure 4a–d exclude duplexes, bulges, internal loops, and multi-branched loops. As shown in Figure S8a–d, all consensus domains involve bases from a fixed base segment at either the 5′ or 3′ end—as high as 100% in consensus domain 1 for dominant + suboptimal aptamer self-hybridized structures, as indicated in Table S3b in supplementary materials.

Identical SSE, namely single-stranded segments, occur in first consensus domain near the 5’ end in Figure 4a–d. For example, the SSE composition of consensus domain 1 is “SSSSSSSS” in the dominant AuNR aptamers and “SSSSSS” in the dominant random sequence in Figure 4a,c. This composition of these first consensus domains is not surprising given the prominence of single-stranded segments near the 5’ end in both PSSM plots in Figure 3a,b. Subsequent consensus domains, however, generally exhibit more diversity in compositions. For example, the structural element composition of consensus domain 6 in the dominant aptamer structures in Figure 4a includes a series of hairpin loops, hairpin stems, and single-stranded segments. Compositional differences in the last consensus domain of all four sets of MSS$A closest to the 3’ end in Figure 4a–d appear more striking, considering the fact that at least 84% of the SS$ in these last consensus domains occur in the fixed base segment, as indicated in Table S3 in supplementary materials. For this fixed base segment near the 3’ end, position-dependent mappings (Figure 1) and matrices (Figure 2) of SSE show the prominence of a previously discussed hairpin stem/loop at bases 55–67. The frequent occurrence of this particular hairpin with the three-adenine loop near the 3’ end in the aptamers may serve as a structural “anchor” in subsequent global alignment analysis. While truncation experiments are beyond the scope of the current work, future screening efforts can either intentionally include portions of this hairpin loop (e.g., in the central variable segment) or intentionally avoid these potential structural anchors by employing libraries consisting of duplex-flanked sequences, in which only the central variable yields various self-hybridized structural elements.

## 3. Conclusions

One can describe aptamer screening efforts via SELEX as a black box of *in vitro* experiments lacking design rules for including promising binding motifs within the screening library. In the absence of any design rules, one must screen through large random sequences to first identify promising candidates for a particular target. In an effort to shine light on these black box experiments, aptamers in this work were identified using CompELS and then analyzed and compared to a background of random sequences. Using a classification scheme to break down predicted secondary structures into smaller elements, this work identified features ranging from prominent individual structural elements at particular base positions to multiple structural elements defining a consensus domain among aligned sequences. Such analysis can enable data mining of first-generation aptamers emerging from random sequence libraries to inform a rational design approach for subsequent libraries to find better second-generation aptamers. As just one example, multi-branched loops found in a majority of base positions in large random sequence populations were relatively rare in the aptamers. Thus, informed by these comparative studies, the next screening libraries for the same target or for a related target could intentionally incorporate promising shared motifs found in this first generation of aptamers (e.g., include adenine-rich loop in the central segment) or intentionally exclude specific elements absent among the first generation of aptamers (e.g., avoiding the likelihood of multi-branched loops by using shorter candidate sequences). In addition to applying these analytical tools to self-hybridized DNA aptamers, the analytical approaches reported here can be expanded to evaluate genomically relevant single-stranded DNA segments that arise during cell processes such as replication and DNA repair. Finally, combining CompELS with these analytical structural tools to find the best aptamer candidates from designer libraries may help inform subsequent experimental validation of self-hybridized aptamers, alone and bound to target species.

## 4. Materials and Methods

### 4.1. Materials

DNA screening libraries were comprised of 69 base-long template strands with a central 40 base randomized segment flanked by constant or fixed sequence segments necessary for primer binding during PCR (5’-GGG ACA GGG CTA GC-[40N]-GAG GCA AAG CTT CCG-3’). Equibase (25% A, 25% C, 25% T, 25% G) and A-rich (40% A, 20% C, 20% T, 20% G) template strands were synthesized via hand-mixing and purified by the manufacturer (Integrated DNA Technologies, Coralville, IA, USA). The motivation for using A-rich screening libraries stems from prior work indicating stronger interactions between gold and adenine bases [60,61]. Reverse primer (5’-CGG AAG CTT TGC CTC-3’), phosphorylated reverse primer (5’-Phos-CGG AAG CTT TGC CTC-3’), and forward primer (5’-GGG ACA GGG CTA GC-3’) were also purchased from and HPLC purified by IDT.

The dNTP mix (10 mM), P/C/I or phenol:chloroform:isoamyl alcohol (25:24:1), ethidium bromide, TOPO TA Cloning Kit for Subcloning, One Shot^®^ TOP10 Chemically Competent *E. coli*, and X-gal were purchased from Invitrogen (Grand Island, NY, USA). GoTaq DNA polymerase and 5X colorless GoTaq reaction buffer were purchased from Promega (Madison, WI, USA). CaCl_2_, HEPES, MgCl_2_, CaCl_2_, and KCl were purchased from BDH Chemicals (VWR Scientific, Radnor, PA, USA). S.O.C. medium, ethanol, and Tris EDTA pH 7.4 were purchased from Fisher Scientific (Pittsburgh, PA, USA). Hexadecyltrimethylammonium bromide, gold (III) chloride hydrate, silver nitrate, L-ascorbic acid, ampicillin sodium salt, sodium borohydride, and dimethylformamide were purchased from Sigma Aldrich (St. Louis, MO, USA). Agar (bacteriological), glycogen, and LB broth (Luria-Bertani) were purchased from Amresco (Solon, OH, USA). Lambda exonuclease enzyme and 10X lambda exonuclease reaction buffer were purchased from New England Biolabs (Ipswich, MA, USA). The MinElute PCR Purification Kit was purchased from Qiagen (Gaithersburg, MD, USA). The pH 7.4 aptamer binding buffer (ABB) used for CompELS-based selection consisted of 20 mM HEPES, 2 mM MgCl_2_, 150 mM NaCl, 2 mM CaCl_2_, and 2 mM KCl. All buffers and synthesis were prepared using 0.2 µM filtered water (18 MΩ-cm) from a Barnstead Nanopure Ultrapure water purification system (Barnstead, Thermo Fisher Scientific, Inc., Pittsburgh, PA, USA).

### 4.2. Synthesis of Gold Nanorods (AuNR)

A seeded gold nanorod (AuNR) synthesis approach was undertaken to prepare target suspensions [53,62]. A CTAB solution (5 mL, 0.2 M) and a HAuCl_4_ solution (5 mL and 0.5 mM) were mixed with a magnetic stirbar for 30 min at room temperature to feed a seed solution. At the 30 min time point, a fresh solution of 10 mM NaBH_4_ was prepared in ice cold water, and 600 μL was added to the seed solution. The magnetic stir speed was increased to high for 2 min, allowing the solution to change to brownish yellow. For the nanorod growth solution, a CTAB solution (20 mL, 0.2 M) was mixed with a freshly prepared AgNO_3_ solution (640 μL, 5 mM) and then a HAuCl_4_ solution (20 mL, 1 mM) under gentle magnetic stirbar mixing conditions for 2 min. Ascorbic acid (216 μL, 0.1 M) was then added to the nanorod growth solution and continuously stirred until the solution became colorless. Seed solution (48 μL) was then added to the nanorod growth solution and mixed with a magnetic stirbar for 40 min. To inhibit further growth or aging of the AuNR and to remove excess CTAB from solution, the nanorod solution was centrifuged at 21,100× *g* for 30 min, followed by supernatant removal, and AuNR resuspension in 40 mL of nanopure water to complete one wash step. This wash step was repeated once more for a total of two wash steps. Twice-washed nanorods were aged for 3 days at room temperature in preparation for CompELS aptamer screening.

### 4.3. Preparation of ssDNA Library for CompELS Screening

Random sequences were amplified via polymerase chain reactions with either the equibase or A-rich template sequences (0.17 pM), dNTPs (0.2 mM), forward primer (60 nM), reverse primers (60 nM), GoTaq polymerase (0.05 U/μL), and 1X supplied colorless GoTaq buffer. PCR was carried out on a G-Storm thermocycler with a 100 °C heated lid with a 2 min hold at 95 °C followed by 25 PCR cycles (30 s denaturation at 95 °C; 30 s annealing at 47 °C; 30 s extension at 72 °C), and a final hold at 4 °C. An ethanol precipitation was performed on the resultant PCR product. Resuspended PCR product was digested with lambda exonuclease at 5 U/μg following the manufacturer’s instructions to remove the phosphorylated hybridization partners. P/C/I extraction was performed on the digested PCR product and followed with another ethanol precipitation. Final ssDNA product was resuspended in aptamer binding buffer (ABB) and ssDNA concentration was adjusted to 2.5 μM and stored at 4 °C until used for screening.

### 4.4. Competition-Enhanced Selection of Ligands (CompELS) Screening for DNA Aptamers against AuNR Targets

Aptamer selection was performed in three separate CompELS sessions against the AuNR using ssDNA random libraries with equivalent 25% distribution in bases for the first two screenings (sequence sets 1XX and 2XX) and using an A-rich library for the third screening (sequence set 4XX). The prepared ssDNA library was separated into 10 aliquots of 100 μL in PCR tubes and denatured in the thermocycler with heated lid (100 °C); 90 °C for 10 min; 4 °C for 15 min; and 24 °C for 5 min. Then, 142 μL of 2X washed AuNR were aliquoted into a PCR tube and centrifuged at 21,100× *g* for 30 min and the supernatant was removed (wash step). To minimize potential nonspecific binding of ssDNA template strands, AuNR were resuspended in 100 μL of 2 μM dNTPs in ABB and incubated for 30 min on a rotomixer. After the 30 min incubation with dNTPs, another wash step was performed. At this point the AuNR had irreversibly aggregated into a visible pellet. A single aliquot of ssDNA library was added to the AuNR, incubated for 30 min on rotomixer, and followed by a wash step. This incubation-wash series was repeated until all 10 library aliquots had been incubated with AuNR target. Following the last of 10 target-library incubation and wash cycles, 200 μL of ABB was added to the AuNR suspension followed by centrifugation and supernatant removal. These wash steps with ABB were repeated for a total of six washes. Following the six washes, nanopure water (74.7 μL), 5X GoTaq buffer (20 μL), dNTPs (2 μL, 10 mM), nonphosphoylated reverse primer (1.2 μL, 5 μM), forward primer (1.2 μL, 5 μM), and GoTaq (1 μL, 5 U/μL) were added to the AuNR. PCR cycling was carried out (in the presence of AuNR target) as detailed previously for the ssDNA library preparation, and dsDNA PCR product was stored at 4 °C until purification for vector insertion steps were undertaken, as described next.

A Qiagen (Gaithersburg, MD) PCR purification kit was used, and purified product was resuspended in 10 μL of 10 mM Tris-HCl pH 8.5. The 10 μL of purified PCR product was placed on ice and salt solution (2.5 μL, 1.2 M NaCl, 10 mM MgCl2) and 2.5 μL TOPO vector from TOPO TA Cloning Kit (Invitrogen, Life Technologies, Grand Island, NY) was added. This ligation mixture was placed on a thermomixer at room temperature for 15 min at 500 rpm. Then, 5 μL of the ligation mixture was added to the TOP10 cells, gently mixed, and put on ice for 2 h. TOP10 cells and ligation mix were then heat shocked at 42 °C for 30 s. Next, 250 μL of SOC (super optimal bath) medium was added to each vial. Resulting cell suspensions were incubated on a shaker table incubator for 1 h 37 °C and 250 rpm. Transformed bacteria were then plated on LB-agar medium supplemented with ampicillin and X-gal, followed by overnight growth in a 37 °C incubator. Twenty-one positive bacterial colonies resulting from CompELS using each of two screening libraries (i.e., normal or A-rich random ssDNA libraries) were randomly picked. After standard plasmid purification following the manufacturer’s directions, samples were sent to GENEWIZ Inc. for sequencing analysis (South Plainfield, NJ, USA).

### 4.5. Primary Structure Analysis of DNA Aptamers

The primary sequences of identified aptamer candidates were analyzed and aligned using T-Coffee multiple sequence alignment of the 40-base central randomized regions (bases 15–54) of the sequences to identify and compare position-dependent bases among all aptamers selected from both the normal and A-rich libraries. T-Coffee analysis was carried out online using default settings (http://www.ebi.ac.uk Accessed: 4/27/17). Alignment results were inserted into Microsoft Excel 2016 and analyzed and color-coded using a macro. MEME 4.9.1 (freely accessed in 7/20/2017 at http://ebi.edu.au/ftp/software/MEME/) was then used to identify position-independent sequence segment motifs that occur anywhere within the central 40 base variable segments of the aptamer sequences [63,64].

### 4.6. Predictions of Dominant and Suboptimal Secondary Structures for DNA Aptamer Sequences and Random DNA Sequences

Zuker’s mfold web server [65] was used to generate secondary structures of all sequences under conditions mimicking that of the aptamer binding buffer (ABB) of [Na^+^] = 152 mM, [Mg++] = 4 mM at 23 °C and to calculate the difference in Gibbs free energy, *dG*, of self-hybridization. The secondary structure of a given sequence with the lowest predicted *dG* value is designated with a nomenclature extension of S1 (e.g., 101.S1) to identify it as the most thermodynamically favorable or *dominant* secondary structure. Other secondary structures of the same sequence whose *dG* was within 5% of the dominant secondary structure’s *dG* were also considered for analysis. These additional secondary structures are referred to as *suboptimal* secondary structures with an assigned nomenclature extension of S2 through S3 (e.g., 101.S2) in order of the next most favorable secondary structure (i.e., second most negative *dG* value) to least favorable secondary structure (i.e., least negative *dG* value). In addition to carrying out secondary structure analysis for the 42 AuNR aptamers resulting from CompELS screening, this same analysis was also carried out for 1000 random DNA sequences in order to generate a background for analytical purposes. While both the aptamers and random sequences share the same fixed base segments as 5’-GGG ACA GGG CTA GC-[40N]-GAG GCA AAG CTT CCG-3’, the 40N segment for the random DNA sequence populations were generated using the built-in RAND function in Microsoft Excel 2016 [66,67].

### 4.7. Defining Secondary Structure Elements (SSE), Secondary Structure Families (SSF), and Secondary Structure Strings (SS$) for Aptamers and Random DNA Sequences

Each secondary structure for a given sequence was broken down into distinct secondary structure elements (SSE) illustrated in Scheme 2, namely, single-stranded segment(s), hairpin stem(s)/loop(s), internal loop(s), bulge(s), duplex(es), and multi-branched loop(s). Two or more aptamer sequences were then assigned to the same secondary structure family (SSF) if they share the exact same numbers of each unique SSE. SSF assignments did not take into consideration the base content, base length, or base position of a given SSE.

The next set of analytical endeavors entailed converting each self-hybridized sequence into text string or secondary structure string (SS$). Since DNA primary structure consists of four bases, most sequence analysis tools limit input to a four-letter alphabet (corresponding to A, T, C, and G bases). In order to use ClustalW to identify any global patterns among SS$ sets, the format of the sequences was listed as amino acid residues instead of nucleic acid bases. The one-letter abbreviations for each SSE shown in Scheme 2 corresponds to the one-letter amino acid substitution used in this analysis. For example, each hairpin stem in a self-hybridized sequence was substituted with a histidine residue in ClustalW. Each SSE was thus assigned to a unique amino acid residue (S, H, L, I, G, D, or M) for converting secondary structures into SS$. The full-length SS$ (69 letters long) was used to map SSE as a function of base positions. These SSE mappings, in turn, were then used to generate position-specific score matrices (PSSM), as detailed in the next subsection.

### 4.8. Using SS$ to Generate Position Specific Score Matrices (PSSM)

In order to score each SS$ for a given sequence secondary structure, the position specific score matrix (PSSM) for a particular SS$ of a sequence *i* over each base position *j* was calculated using Equation (1) as follows:

(1) $$S_{k,j}=H_{j}W_{k,j}$$

in which

$$H_{j}=\frac{log_{2}\left(7\right)}{\sum_{columns\;j}log_{2}\left(\frac{1}{\mu_{k,j}}\right)\left(\frac{1}{N}\right)\sum_{i=j}^{N}\left(X_{i.j}=k\right)}$$

and

$$W_{k,j}=\frac{1}{N}log_{2}\left(\frac{\theta_{k,j}}{\mu_{k,j}}\right)\overset{N}{\underset{i=1}{\sum }}I\left(X_{i.j}=k\right)$$

where

$$\mu_{k,j}=\frac{1}{N}\overset{N}{\underset{i=1}{\sum }}I\left(X_{i.j}=k\right)$$

and

$$\theta_{k,j}=\frac{7\alpha \mu_{k,j}+\sum_{i=j}^{N}I\left(X_{i.j}=k\right)}{7\alpha +N}$$

where *i* ∈ (1, …, *N*)*, j* ∈ (1, …, *l*), *k* ∈ (*S,H,L,I,G,D,M*); *i* is each 69 position-long SS$ in a sequence population comprised of *N* dominant or *N* dominant + suboptimal SS$; *j* is the base position from 1 to length *l* (up to base position 69); *X_i,j_* is the SS$ with an SSE identity of *X* at base position *j*; *k* is the one-letter abbreviation for SSE (S, H, L, I, G, D, or M); *I(X_i,j_ = k)* is an identity matrix of the seven SSE with a value of 0 (if SSE identities do not match) or 1 (if SSE identities do match); *μ_k,j_* is the background position frequency matrix; *θ_k,j_* is the normalized position probability matrix; *α* is the additive smoothing parameter; *H_j_* is the normalization matrix; and *W_k,j_* is the position weight matrix.

### 4.9. Using SS$ to Perform Multiple Secondary Structure String Alignment (MSS$A)

While ClustalW in MEGA7 [68] is protein sequence alignment program, ClustalW is employed in the current work to globally align SSE found in dominant or dominant + suboptimal self-hybridized structures in DNA aptamers to compare to analogous alignments for a random sequence population. To distinguish this form of analysis from PSSM described above, this global alignment is referred to as multiple secondary structure string alignment (MSS$A) in the current work. To prevent artifacts that can arise from default settings that allow amino acid substitutions (e.g., substituting one acidic residue such as aspartic acid with glutamic acid) an identity matrix was used as the substitution matrix for alignment along with removal of residue-specific penalties and hydrophilic penalties. Otherwise, the default setting for all other field options in MEGA7 were employed using version 7.0.26 during the date range of 7/4/2017-7/25/2017. Data were exported into MATLAB 2016 for analysis by developed program algorithms, and output was charted using Microsoft Excel 2016.

### 4.10. Defining Secondary Structure Consensus Domains in MSS$A with Gaps Included

Following MSS$A analysis of each group of sequences, various consensus domains, gap regions, and non-gap regions were identified and numbered, as described next. Consensus domains consisting of a SS$ with ≥50% consensus were identified. In addition to consensus domains, regions within MSS$A containing ≥50% inserted gaps were identified and numbered as *gap regions*. Regions consisting of at least 50% SSEs, but lacking sufficient consensus were identified and numbered as *non-gap regions*. After covering these possible categories of alignment regions, to display large datasets succinctly, 2D global alignment maps were “compressed” into 1D lines to more easily distinguish and compare each consensus domain, gap region, and non-gap region for aptamers and random sequences. The following data are also provided for each MSS$A: Average length and standard deviation of length for each domain or region in the alignment; the average number of consensus domains observed per sequence; and the average percentage of SS$ associated with a given consensus domain. For consensus domains, the following additional information is provided: Its SS$ identity; average consensus value across the entire domain (referred to as *conserved*); statistical frequency of observation of a domain in a sequence set (referred to as *frequency*); and the fraction of the domain associated with the fixed base regions (referred to as *fraction fixed base*).

### 4.11. Removing Gaps to Determine Distribution of Consensus Domains in MSS$A

Following MSS$A to identify consensus domains, as described above, gap regions were subsequently removed to determine the base position dependence of each consensus domain. Using algorithms developed in MATLAB 2016, the SS$ associated with each consensus domain was plotted as function of base position to then perform the following analyses: Base position-specific distribution of each consensus domain, average distance from an identified domain *n* to domain *n* + 1 (*d_(n,n+1)_*), average distance from a domain to the closest previously occurring domain or SS$ end 5’ to 3’ (*d_prior_*), average distance from a domain to the next occurring domain or SS$ end 5’ to 3’ (*d_next_*), and average number of bases lost from the central portion of a domain (*domain loss*). Plots of the identified domain distributions were created in Microsoft Excel 2016.

## Figures and Tables

**Table 1:** List of secondary structure family (SSF) group number, structural element composition, aptamer nomenclatures, total number of members, and the numbers of dominant and suboptimal self-hybridized structures for all 11 SSF assigned among all AuNR aptamers identified via CompELS.

SSF No.	Structural Element Composition	Aptamer Nomenclature ^1,2^ of SSF Members	No. of SSF Members	No. Dominant (Suboptimal) Self-Hybridized Structures
SSF1	2S 2H/L 1I 0G 1D 0M	111.S1, 116.S1, 116.S2	3	2 (1)
SSF2	2S 2H/L 1I 1G 2D 0M	101.S1, 117.S1, 418.S1	3	3 (0)
SSF3	3S 2H/L 0I 0G 0D 0M	111.S2, 111.S3, 120.S1, 214.S1, 403.S1, 412.S2	6	3 (3)
SSF4	3S 2H/L 0I 1G 1D 0M	401.S1, 420.S1	2	2 (0)
SSF5	3S 2H/L 1I 0G 1D 0M	108.S2, 112.S1, 406.S1	3	2 (1)
SSF6	3S 2H/L 2I 0G 2D 0M	110.S1, 115.S1, 416.S1	3	3 (0)
SSF7	4S 3H/L 0I 0G 0D 0M	102.S1, 212.S1, 217.S1, 219.S1, 404.S1, 412.S1	6	6 (0)
SSF8	4S 3H/L 0I 1G 1D 0M	101.S2, 118.S1, 201.S2, 210.S1	4	2 (2)
SSF9	4S 3H/L 1I 0G 1D 0M	105.S1, 108.S1, 114.S1, 119.S1, 204.S1, 204.S2, 402.S1, 413.S1, 415.S2, 421.S1	10	8 (2)
SSF10	5S 4H/L 0I 0G 0D 0M	217.S2, 410.S1, 417.S1, 419.S1	4	3 (1)
SSF11	5S 4H/L 1I 0G 1D 0M	409.S1, 414.S1	2	2 (0)

^1^ Secondary structures of aptamer are designated as dominant (*.S1) or suboptimal (*.S2 or *.S3); ^2^ Identical aptamer sequences in the same SSF are underlined.

## Supplementary Materials

The following are available online. Figure S1: UV-vis spectroscopy of AuNR; Table S1: List of AuNR aptamers; Figure S2: Multiple sequence alignment of aptamers; Table S2: Gibbs Free Energy values of dominant and suboptimal secondary structures of aptamers; Figure S3: Schematics of 11 SSF identified for aptamers; Figure S4: Bar graphs of percentage difference in dominant-only SSE in aptamer and random sequence populations; Figure S5: Bar graphs of percentage difference in dominant + suboptimal SSE in aptamer and random sequence populations; Figure S6: Two-dimensional heat maps of aligned SSE for aptamers; Figure S7: SS$ for aptamer and random sequence populations, Figure S8: Distribution of secondary structure consensus domains determined from MSS$A of aptamer and random sequence populations; Table S3: Numerical information on secondary structure consensus domains determined from MSS$A of aptamer and random sequence populations; Table S4: Additional numerical information on secondary structure consensus domains determined from MSS$A of aptamer and random sequence populations.

## Abbreviations

ABB	aptamer binding buffer
AuNR	gold nanorod(s)
CompELS	competition-enhanced ligands selection
dsDNA	double-stranded DNA
MEME	Multiple EM (Expectation Maximization) for Motif Elicitation
MSS$A	multiple secondary structure string alignment
P/C/I	phenol:chloroform:isoamyl alcohol
PSSM	position specific score matrix(ces)
SELEX	systematic evolution of ligands by exponential enrichment
SOC	super optimal bath
ssDNA	single-stranded DNA
SSE	secondary structure element(s)
SSF	secondary structure family(ies)
SS$	secondary structure string(s)
