# Supplementary material for: Analyzing Secondary Structure Patterns in DNA Aptamers Identified via CompELS
Source: Molecules. 2019 Apr 21;24(8):1572. doi: 10.3390/molecules24081572 (PMC6515186; doi:10.3390/molecules24081572)
Supplement: Supplementary file 1 [file molecules-24-01572-s001.pdf]

Supplementary Materials

"Analyzing secondary structure patterns in DNA aptamers identified via CompELS,"
by Richard Sullivan, Mary Catherine Adams, Rajesh R. Naik, Valeria T. Milam

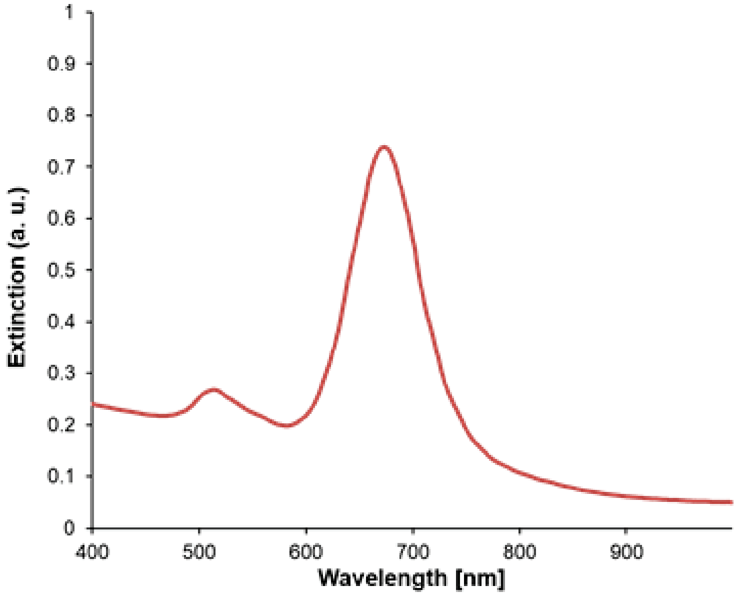

Figure S1. UV-vis spectra of AuNR used for CompELS screening. For spectroscopy studies, AuNR were prepared using a seeded growth, washed two times to remove excess CTAB while maintaining a relatively stable suspension exhibiting peaks values of 513 nm and 674 nm. Prior to the start of CompELS screening, AuNR suspensions were washed an additional (third) time on day 3.

Table S1. List of nomenclatures and central 40 base-long segments of AuNR aptamer candidates identified via CompELS screening using either an (left) equibase screening library yielding 23 AuNR aptamer candidates or (right) adenine-rich random screening library yielding 19 AuNR aptamer candidates. The full-length 69 base-long sequences include two fixed base segments and a central variable region (40N) as follows: 5'-GGGACAGGGCTAGC(40N)GAGGCAAAGCTTCCG-3'.

AuNR Aptamers from Normal Libraries

Aptamer	Sequence (5′→3′)
101	ATATGTAATTATGCGCTTCTAGTTAATAGGCCCTTTGCAA
102	ATTAATTTAAGCTTCATCAGACAAGAGCAGGGGCATACAA
105	ATATGATCGTTTATAAGCTGTTCTTCTTCCATGGTTACAT
108	AGTGTTACAGTGTTGGGTTTTTTAATAACAATGGATTTGT
110	GTTTAATACAGGTTCACGAGGCATTACGTTTACTTACTCT
111	TGCAGCGACGGGTTATTATTCTTGTACAGTTCTTTTTCAC
112	TAATACATAACGTAGTTATTCCGCATTTAACTAGAAAATT
114	ATGATAACCTATTTTGCATTTATATGGTGCAGAACCATTT
115	AACGAAGTCTGGGTAGAGGCGTGATGTGGGATGTCTAGGT
116	ATCTTTCTCGTTTGAAAACCTCGGCTCTTTGCCATGGGCA
117	TAGGGCGTGGGTGGTGTTTAATATTCCCTATGCCCGACGT
118	TATTTGTTGGTCTTTAAAAAGTGTTACACTCGCTATTGCT
119	ACGTAGAGGGGGCTTGTCGTTAACAAAATGGGGTTCTGGC
120	GAAATTAGATCATGCGGCTAAGGGGTTAGTTAAGTATCCA
121	AAAAATTATGATTTGTGAGTGAACTCATTCCCATTGACAT
201	CGTAAATGTCTCTGAACCTTAAACTGTCAGGCAGATTTAT
204	ACGTCGTTGTATGGGACTATTGTAGTAAACTTCAATTATT
207	TAGTTATGGAACGCAATTGGGGGACGGATCTCTTGTCTTT
210	TGCCATTCCGAAACGAAATAAAAGTAACCCTTATTTTCAC
212	CGGGGCTGTTGGGGGCAAATCATTTAGTCATTGAGGTATC
214	GTTATATTTTAAAAGTGTCCATTTGGGCGAATTATGGATA
217	ACCTGATGTTGATGTGTATCCTCCGTCGACAGTATATGGT
219	ATTAGTTTGGTTGTATGTGTTACAGGATTGTAGGGGATGT

AuNR Aptamers from A-Rich Libraries

Aptamer	Sequence (5′→3′)
401	ATATGGATAAGATCTGCAGGCAGAATCCAGTAGTTAATTA
402	ACACGTAAAAAGGTGTGAATCGGTGATGAAGAGGTTTTTC
403	AAACGAAGAGATTATAGATGAGATACCGTCTCGAAACCAC
404	GGCAAATAACTGGACGTACTAAGAACAATAAGCCTGGGTA
405	GGATACGTTAACCAGTACTAAAAGCGAGCATACCTAAAAA
406	GCAAAGAAACTAGGATAAAAGGAGCCAGGATTCACAAATA
409	AAATCGTGAATTTATAGGTATAAACGTGAAATACACAAAT
410	TCTGACAACTTCACTACACGCAACGGGAAAAGATATTGGA
411	CGTTATGAGAAGCAACTTCCTGGCAATAACTAGGAGCCAA
412	AGGTAAGTTATTTGAGATGTCATGTAAGGACAAAGTGGTA
413	TTAACCCGATCACAGTCCGACTCATTAACATATTAGTGTA
414	ATGGATTGCCGAGAACTCCACAAGATCTTAATCGAATGAC
415	AGATAATCAGTCACTAGAAAAAATCCCAGCACCAGGAGTA
416	GATATGACGAAGCAAATGTCATTAATAACAAACAAAGATC
417	AAGGGTAAAATGAATTTAACATAGTATTAGAACGTGTCAC
418	AAGCCTTAAAGGAAACGGACGAAAAAATAAATTAGGTTAT
419	TTTTAGCCATAGAGTACATACGACTCCGGAAAGAATTCAA
420	TGATAATTAAATTATACCCCAGCTATTGTTTACATCGCAC
421	AAAAGAATAAACGACTGAAAAATAAACGGTTAAGAAGTGT

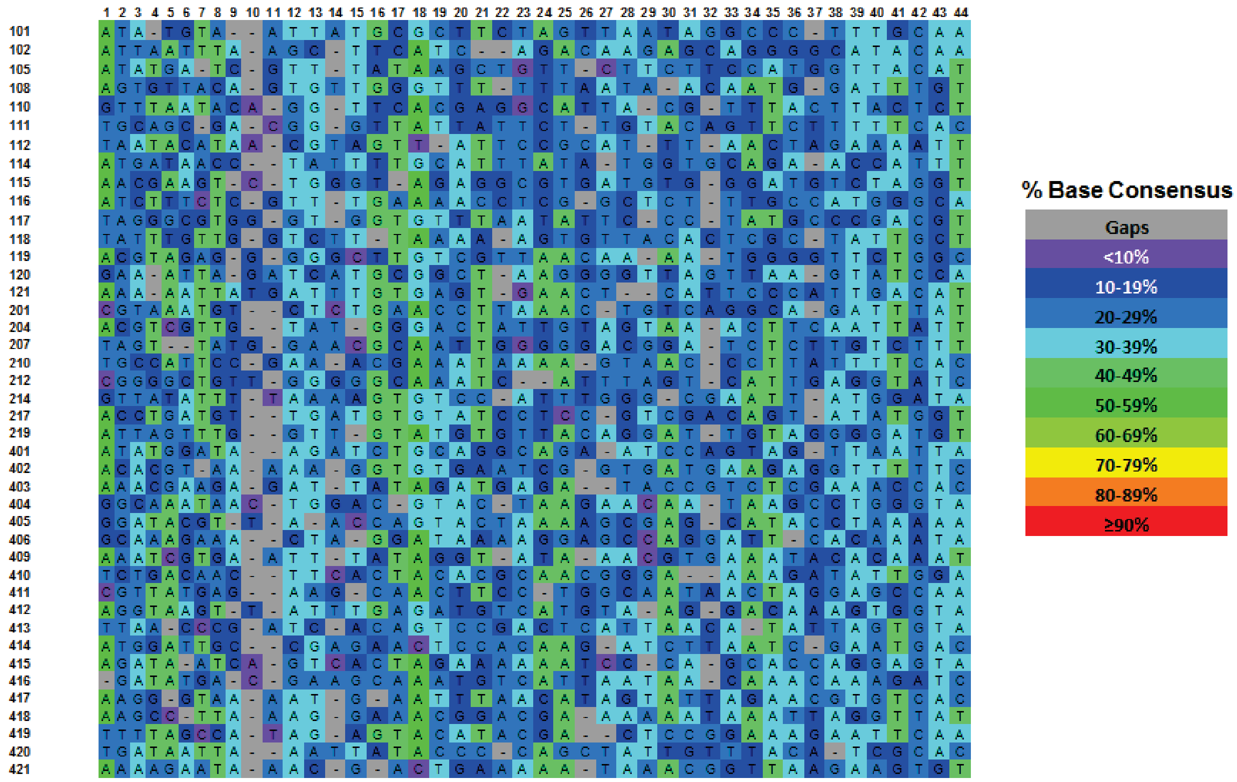

Figure S2. Multiple sequence alignment results for 40-base long central segments of all 42 AuNR aptamer sequences using default settings for DNA in T Coffee (http://www.ebi.ac.uk Accessed: 4/27/17) and color coded in Microsoft Excel 2016. The nomenclature for each aptamer sequence is listed vertically on the left. Each of the four gray dashes corresponds to a nucleotide gap inserted by T Coffee.

Table S2. List of aptamer sequences, differences in Gibbs Free Energy values (dG) of dominant (Structure 1) and suboptimal (Structure 2 and Structure 3) self-hybridized apatmers. Only suboptimal structures within 5% of the Gibbs Free Energy value of the dominant structure are included.

Aptamer Sequence	Structure (S)	dG [kcal/mol]	5% dG Error
**101**	1	-11.17	-0.56
	2	-11.07	
**102**	1	-4.70	-0.24
**105**	1	-6.46	-0.32
**108**	1	-6.48	-0.32
	2	-6.42	
**110**	1	-4.12	-0.21
**111**	1	-3.06	-0.15
	2	-3.02	
	3	-3.00	
**112**	1	-5.23	-0.26
**114**	1	-8.36	-0.42
**115**	1	-4.04	-0.20
**116**	1	-10.54	-0.53
	2	-10.50	
	3	-10.03	
**117**	1	-8.19	-0.41
**118**	1	-7.78	-0.39
**119**	1	-5.69	-0.28
**120**	1	-2.70	-0.14
**121**	1	-6.82	-0.34
**201**	1	-7.07	-0.35
	2	-6.83	
	3	-6.78	
**204**	1	-4.70	-0.24
	2	-4.62	
	3	-4.57	
**207**	1	-6.52	-0.33
**210**	1	-7.31	-0.37
**212**	1	-5.21	-0.26
**214**	1	-4.89	-0.24
**217**	1	-7.52	-0.38
	2	-7.25	
**219**	1	-3.85	-0.19

Aptamer Sequence	Structure (S)	dG [kcal/mol]	5% dG Error
**401**	1	-6.77	-0.34
**402**	1	-8.03	-0.40
**403**	1	-5.08	-0.25
	2	-5.08	
**404**	1	-6.41	-0.32
**405**	1	-7.01	-0.35
**406**	1	-4.72	-0.24
**409**	1	-7.04	-0.35
**410**	1	-5.85	-0.29
**411**	1	-9.08	-0.45
**412**	1	-3.48	-0.17
	2	-3.31	
**413**	1	-4.83	-0.24
**414**	1	-6.89	-0.34
**415**	1	-5.11	-0.26
	2	-5.04	
**416**	1	-6.90	-0.35
**417**	1	-4.61	-0.23
**418**	1	-7.30	-0.37
**419**	1	-10.90	-0.55
**420**	1	-7.31	-0.37
**421**	1	-4.16	-0.21

(a) SSF1: 2S 2H/L 1I 0G 1D 0M

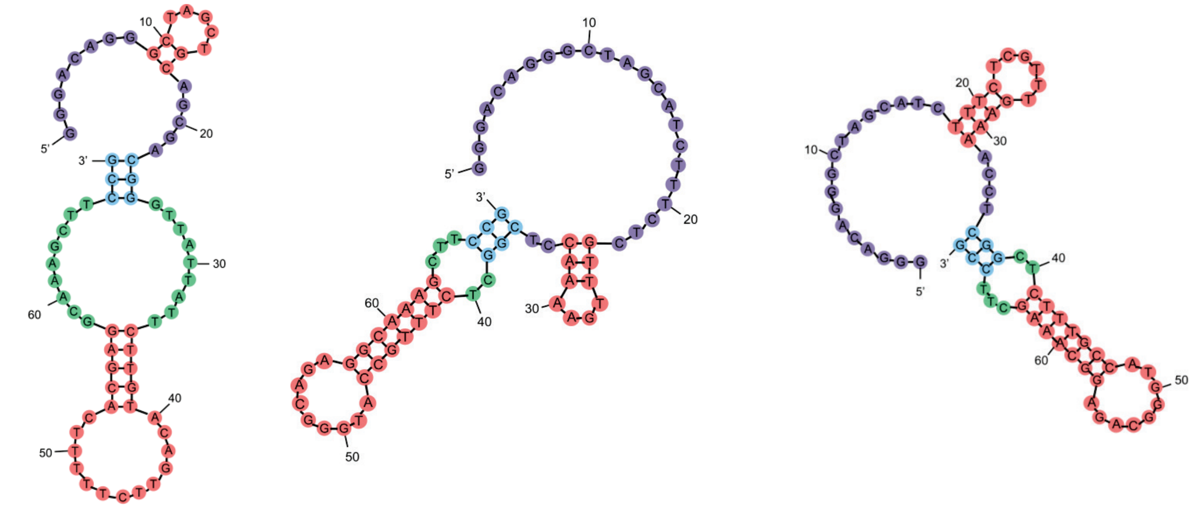

111.S1 116.S1 116.S2

(b) SSF2: 2S 2H/L 1I 1G 2D 0M

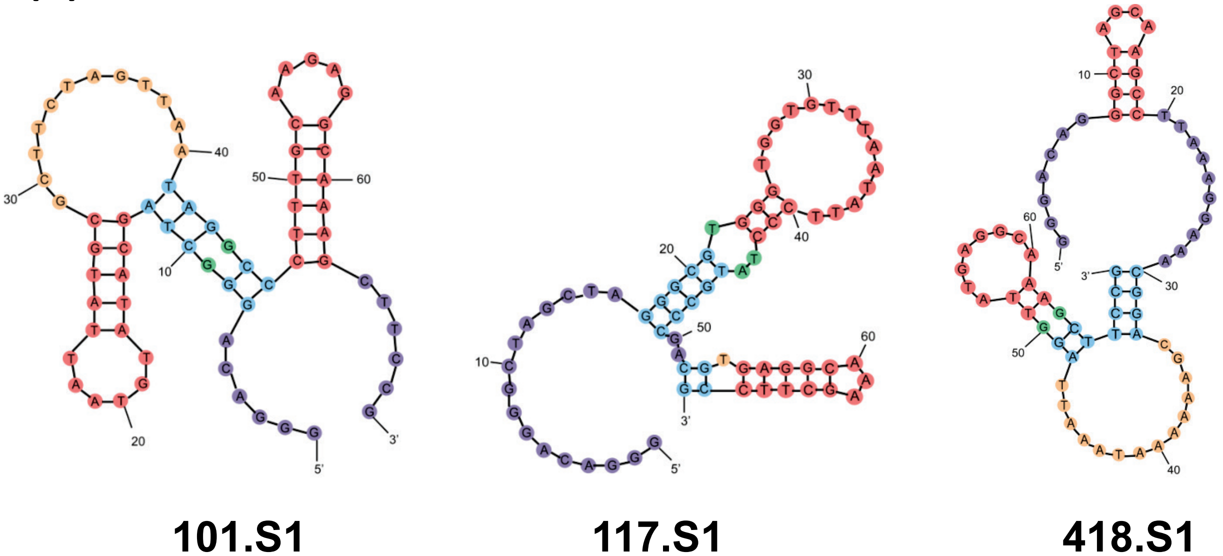

101.S1 117.S1 418.S1

(c) SSF3: 3S 2H/L 0I 0G 0D 0M

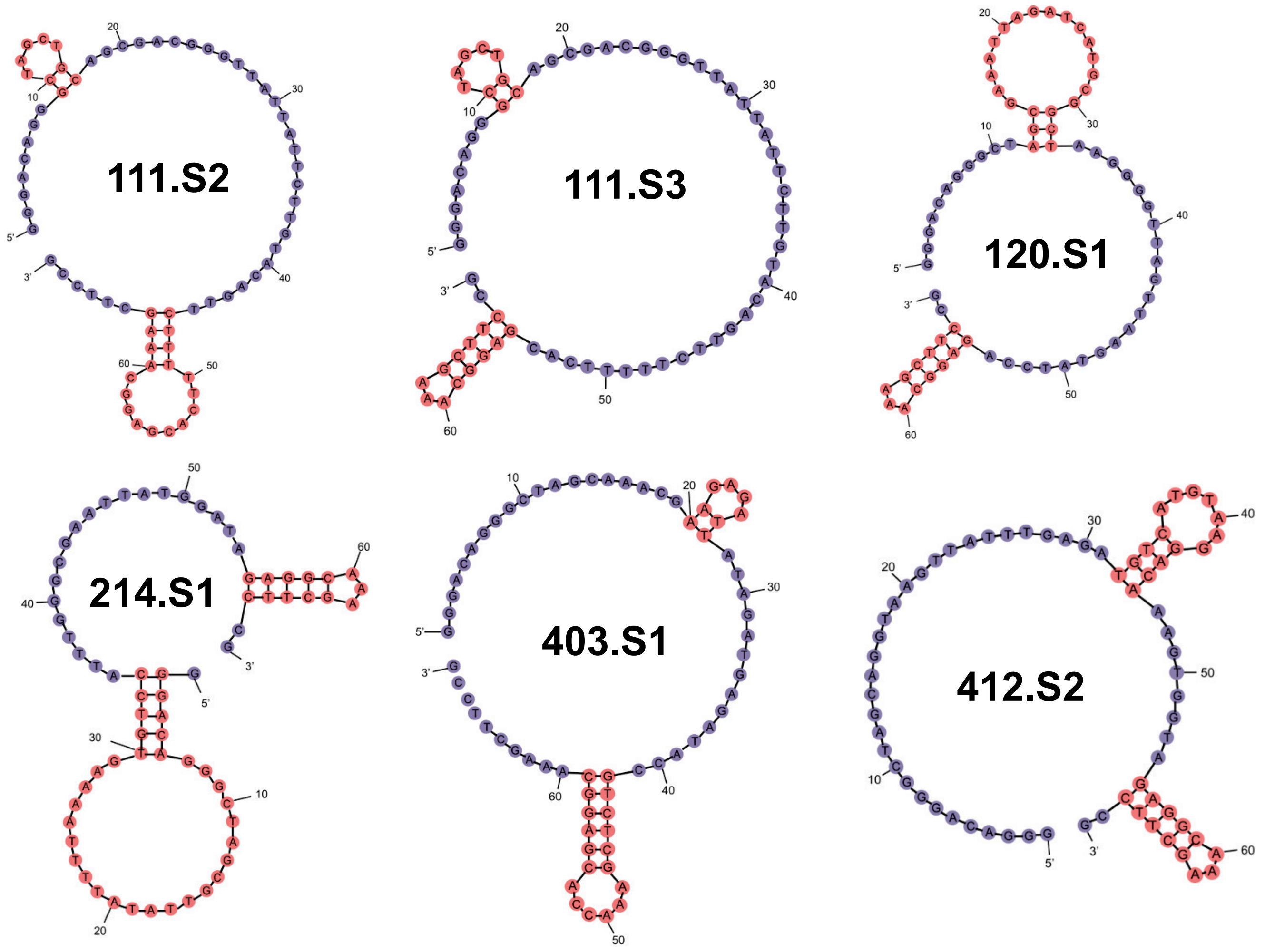

(d) SSF4: 3S 2H/L 0I 1G 1D 0M

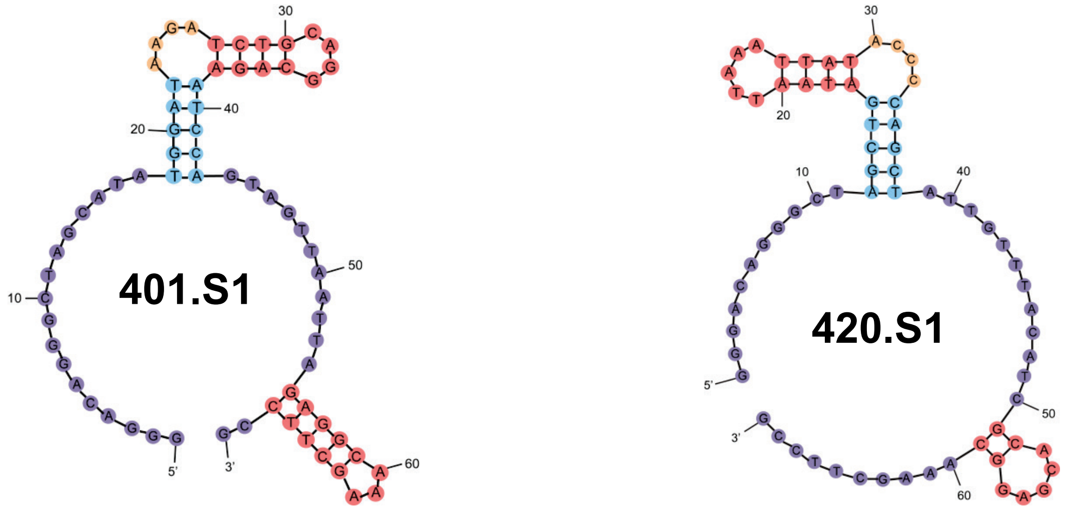

(e) SSF5: 3S 2H/L 1I 0G 1D 0M

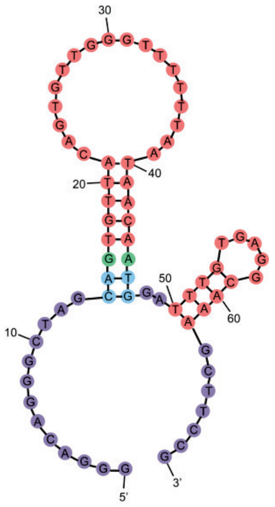

108.S2

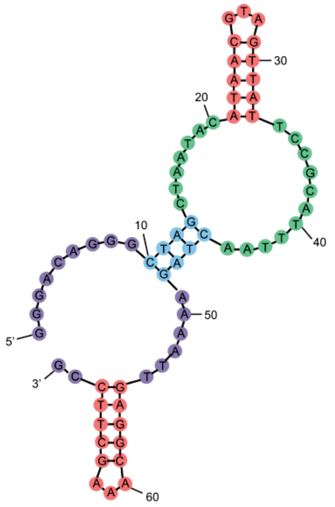

112.S1

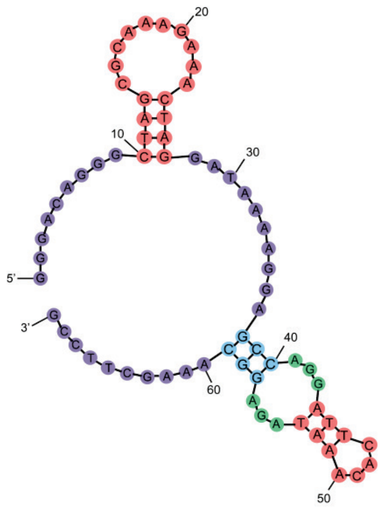

406.S1

(f) SSF6: 3S 2H/L 2I 0G 2D 0M

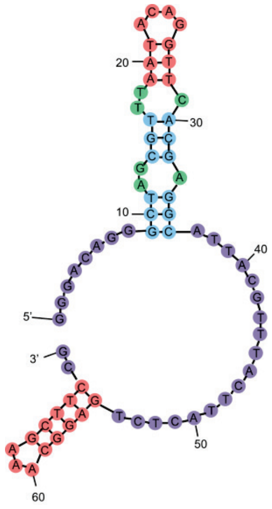

110.S1

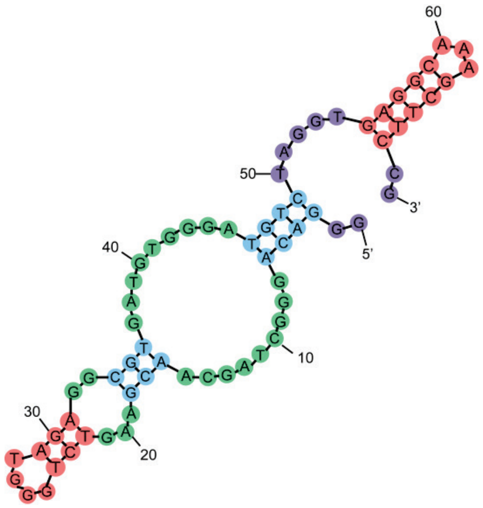

115.S1

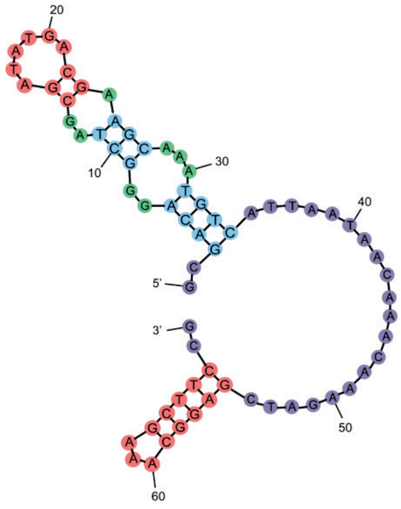

416.S1

(g) SSF7: 4S 3H/L 0I 0G 0D 0M

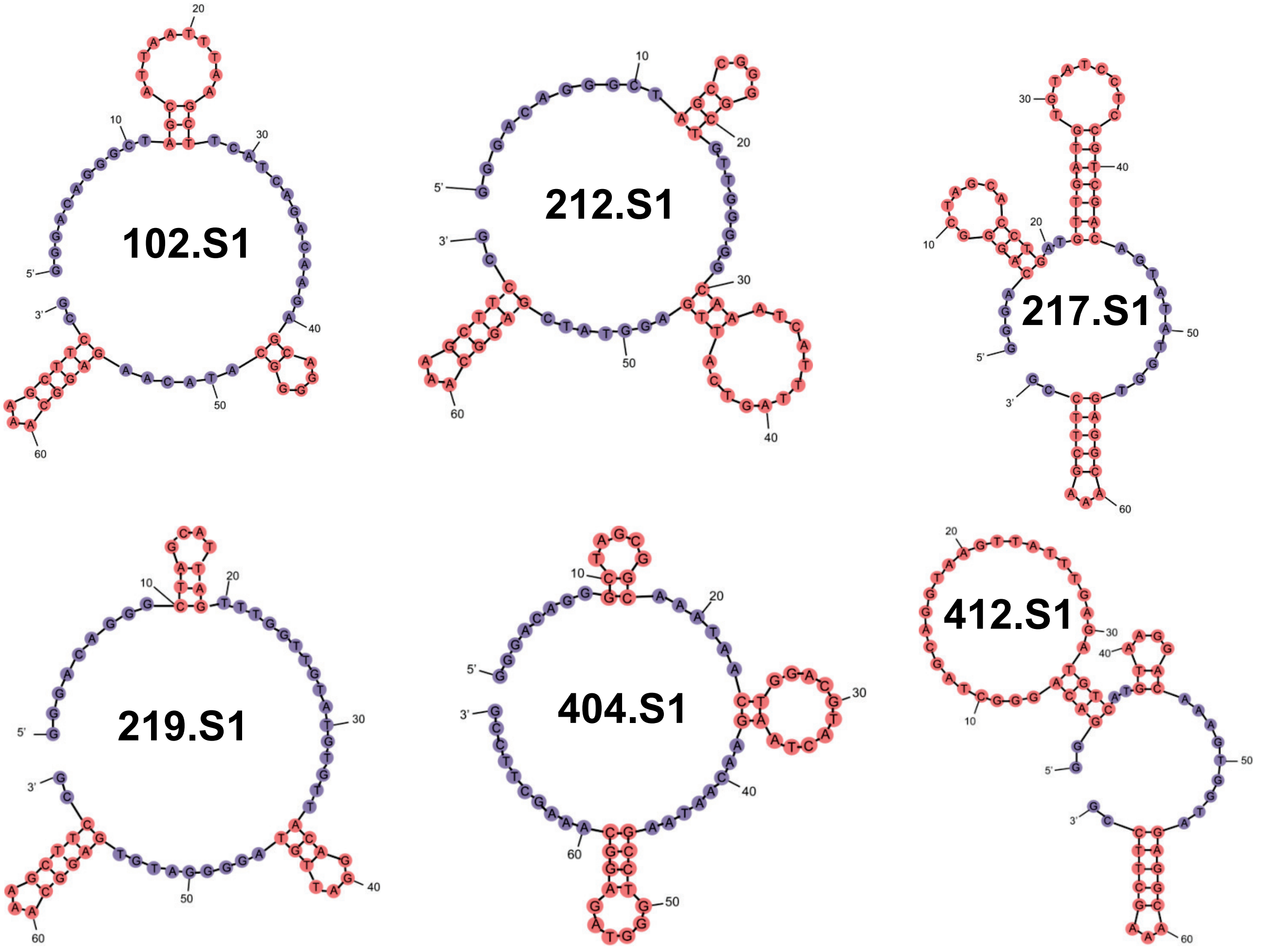

(h) SSF8: 4S 3H/L 0I 1G 1D 0M

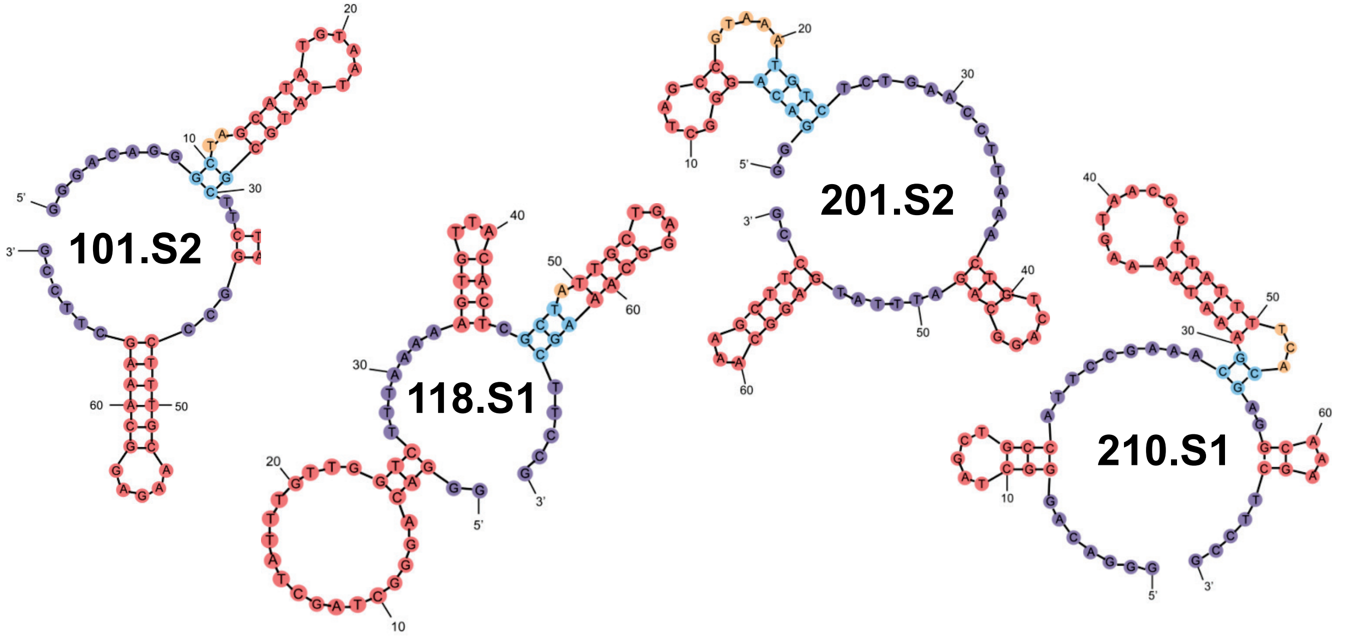

(i) SSF9: 4S 3H/L 1I 0G 1D 0M

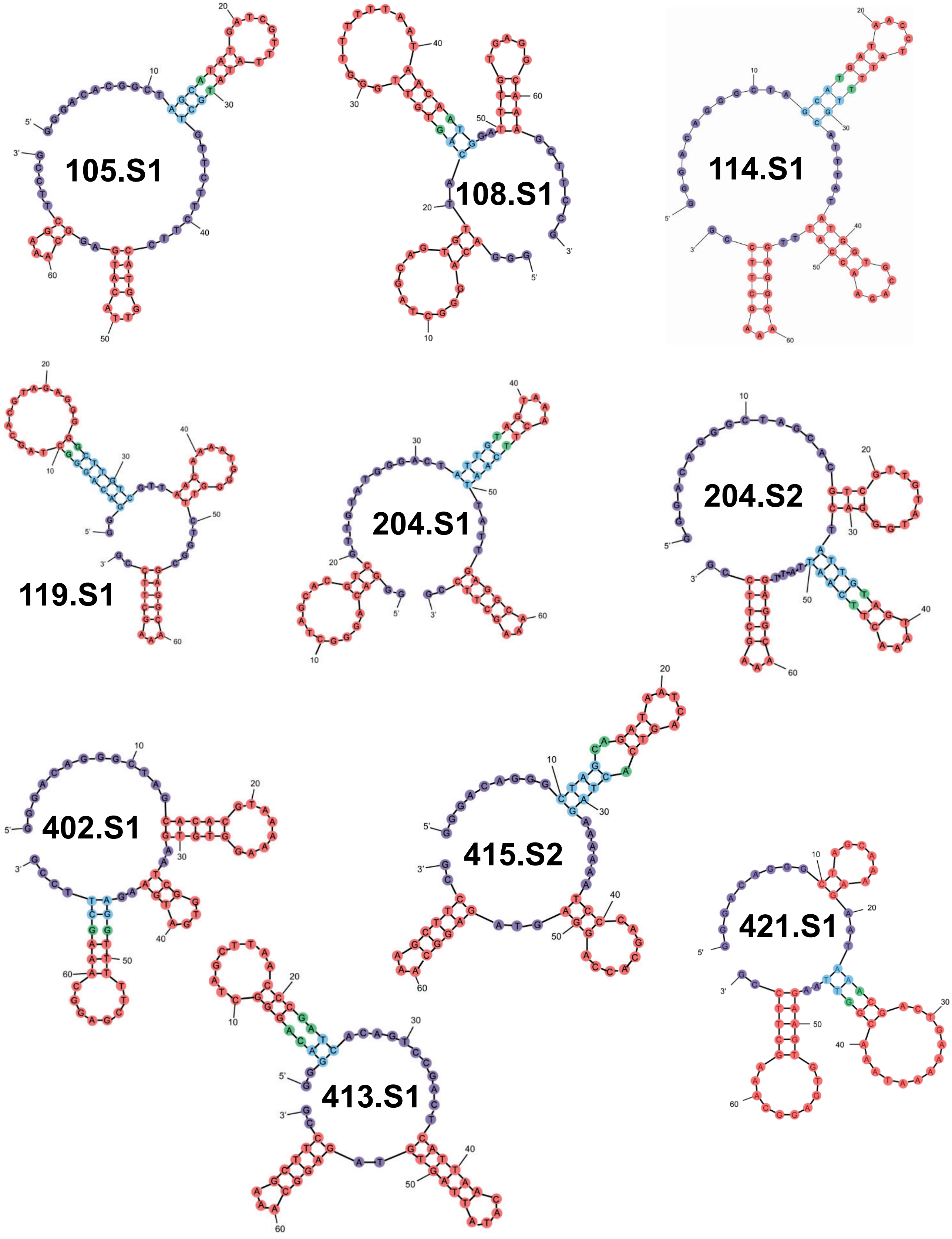

(j) SSF10: 5S 4H/L 0I 0G 0D 0M

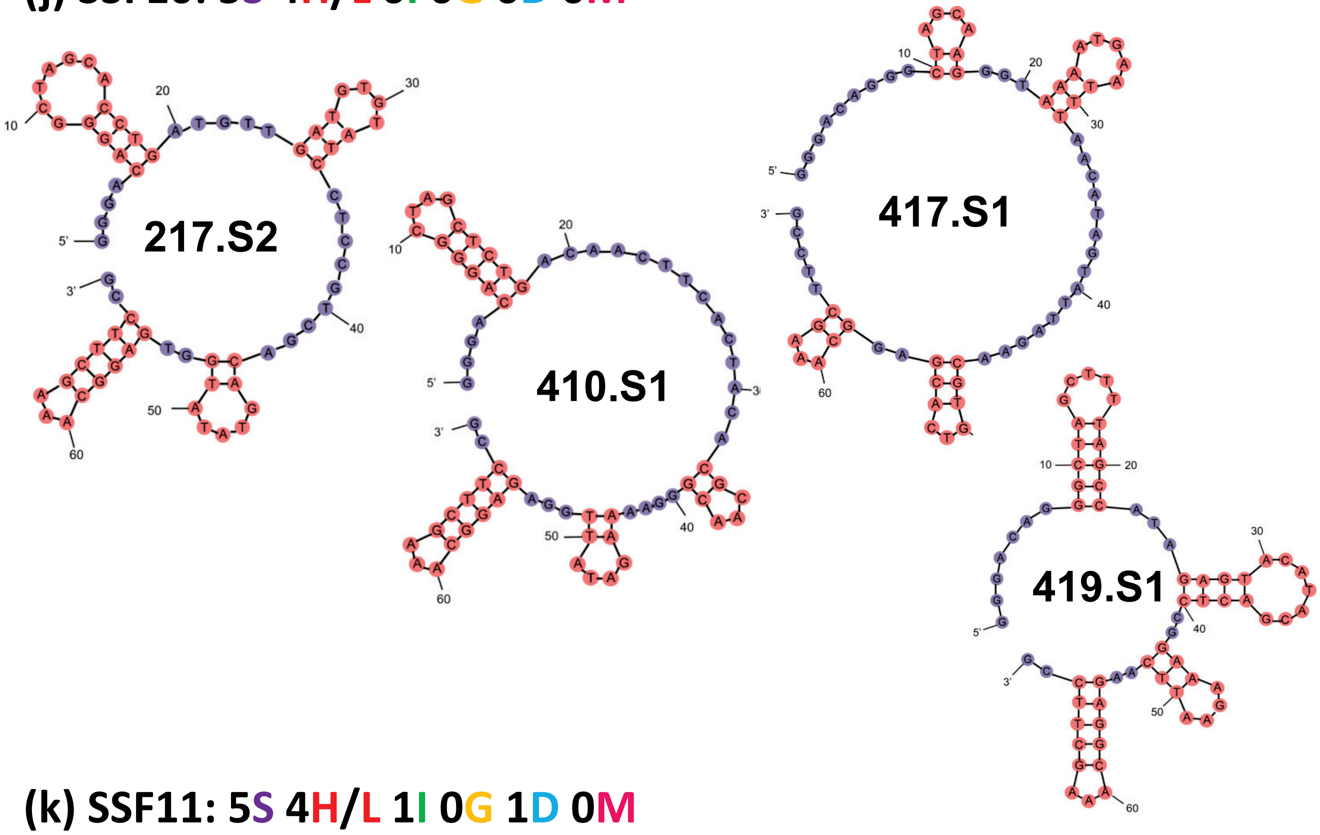

(k) SSF11: 5S 4H/L 1I 0G 1D 0M

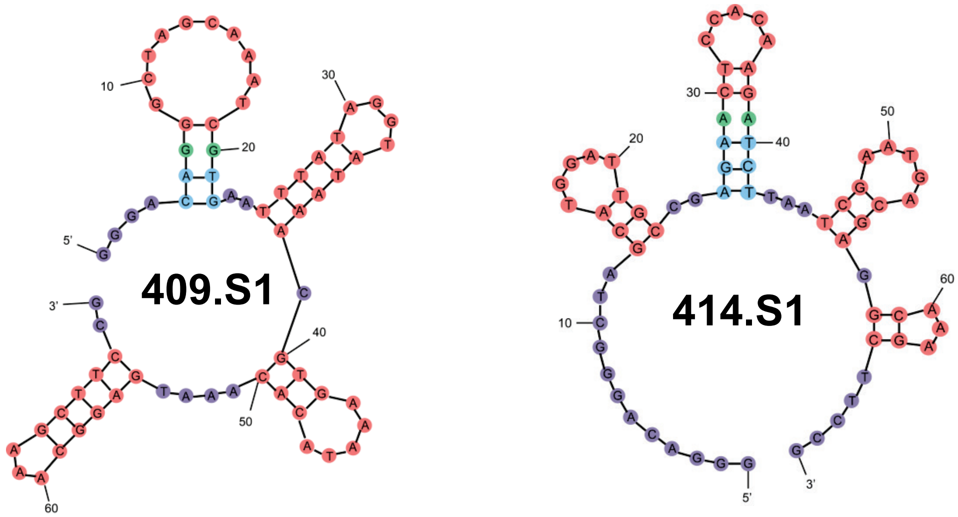

Figure S3. (**a-k**) Schematics of self-hybridized structures of all AuNR aptamer sequence members of the eleven SSF listed in **Table 1**. Each SSE in predicted secondary structures are color-coded as follows: single-stranded segment (purple); hairpin loop and stem (red); internal loop (green); bulge (yellow); duplex (blue); multibranched loop (M).

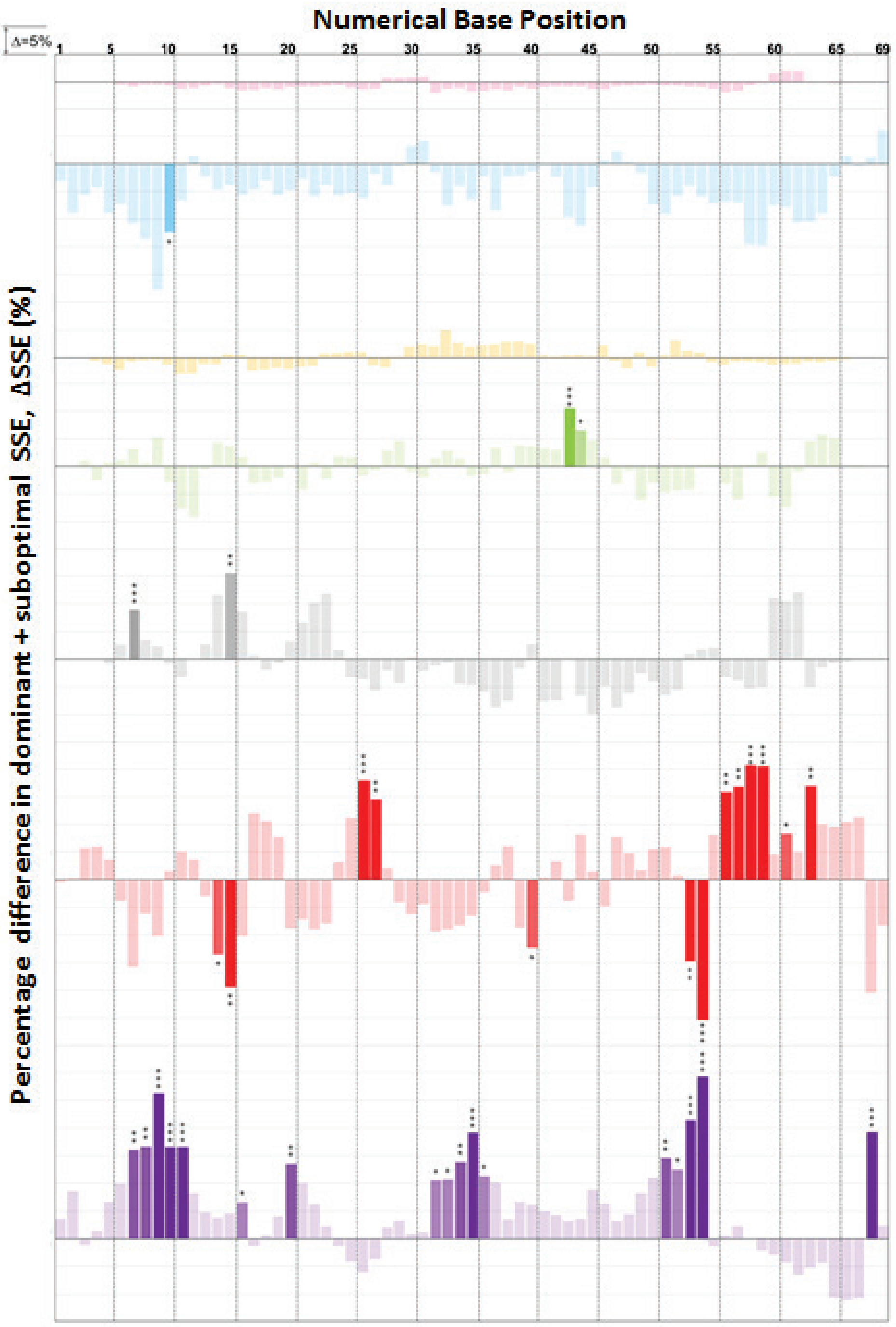

Figure S4. Bar graphs of the percentage difference in dominant-only SSE (ΔSSE (%)) at each numerical base position (1-69) between aptamer and random sequence populations. Each bar resides either above its x-axis (for aptamer sequences exhibiting a positive ΔSSE (%) value) or below its x-axis (for random sequence population exhibiting a positive ΔSSE (%) value). Each bar at a given base position is color-coded as follows: multibranched loops (dark pink); duplexes (blue); bulges (yellow); internal loops (green); hairpin loops (gray); hairpin stems (red); and single-stranded segments (purple). The symbols *, **, and *** series denote a 95%, 97.5%, and 99.5% confidence interval, respectively for significant differences based on a one-tailed 2 proportion Z-test.

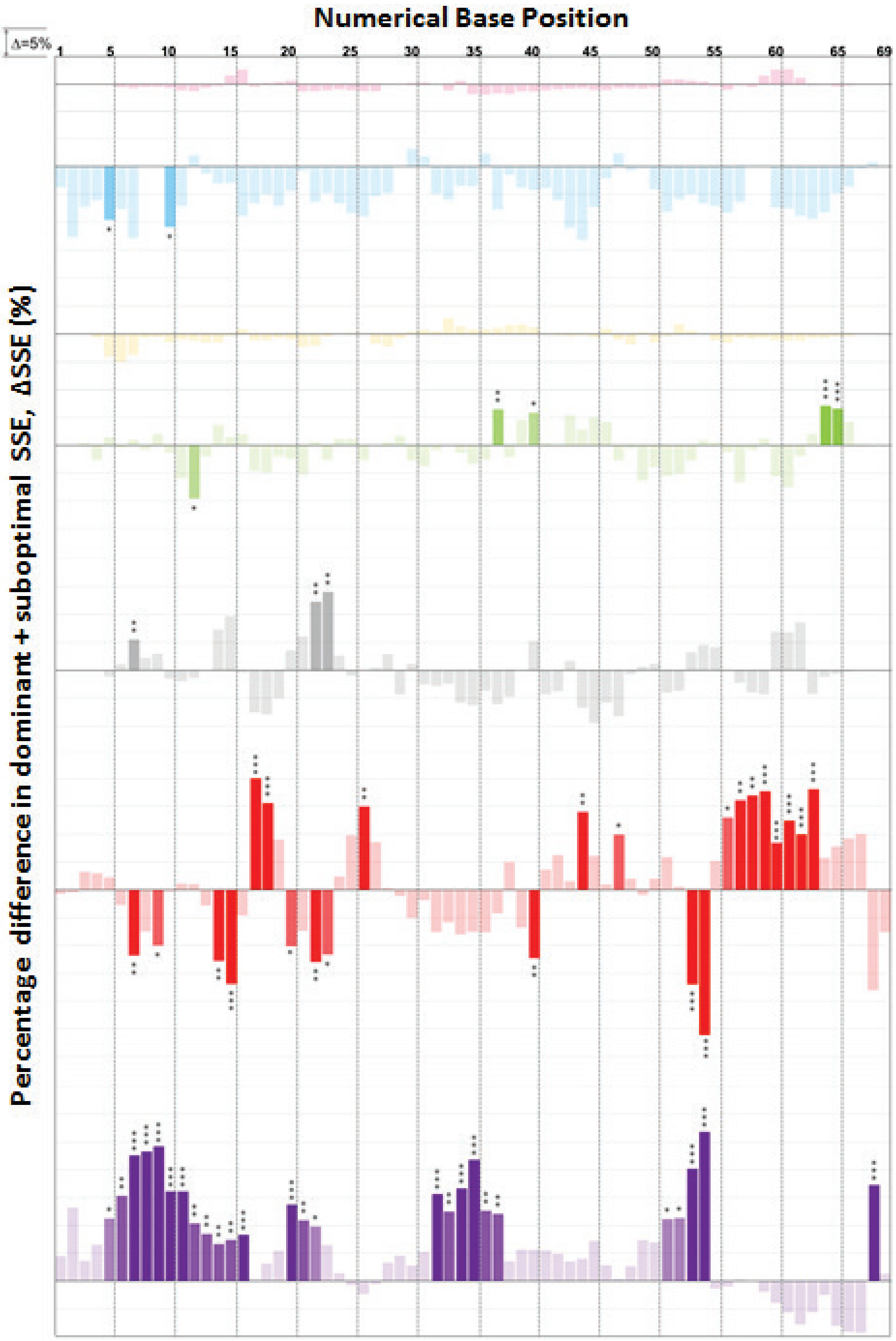

Figure S5. Bar graphs of the percentage difference in dominant + suboptimal SSE (ΔSSE (%)) at each numerical base position (1-69) between aptamer and random sequence populations. Each bar resides either above its x-axis (for aptamer sequences exhibiting a positive ΔSSE (%) value) or below its x-axis (for random sequence population exhibiting a positive ΔSSE (%) value) a given x-axis. Each bar at a given base position is color-coded as follows: multibranched loops (dark pink); duplexes (blue); bulges (yellow); internal loops (green); hairpin loops (gray); hairpin stems (red); and single-stranded segments (purple). The symbols *, **, and *** series denote a 95%, 97.5%, and 99.5% confidence interval, respectively, for significant differences based on a one-tailed 2 proportion Z-test.

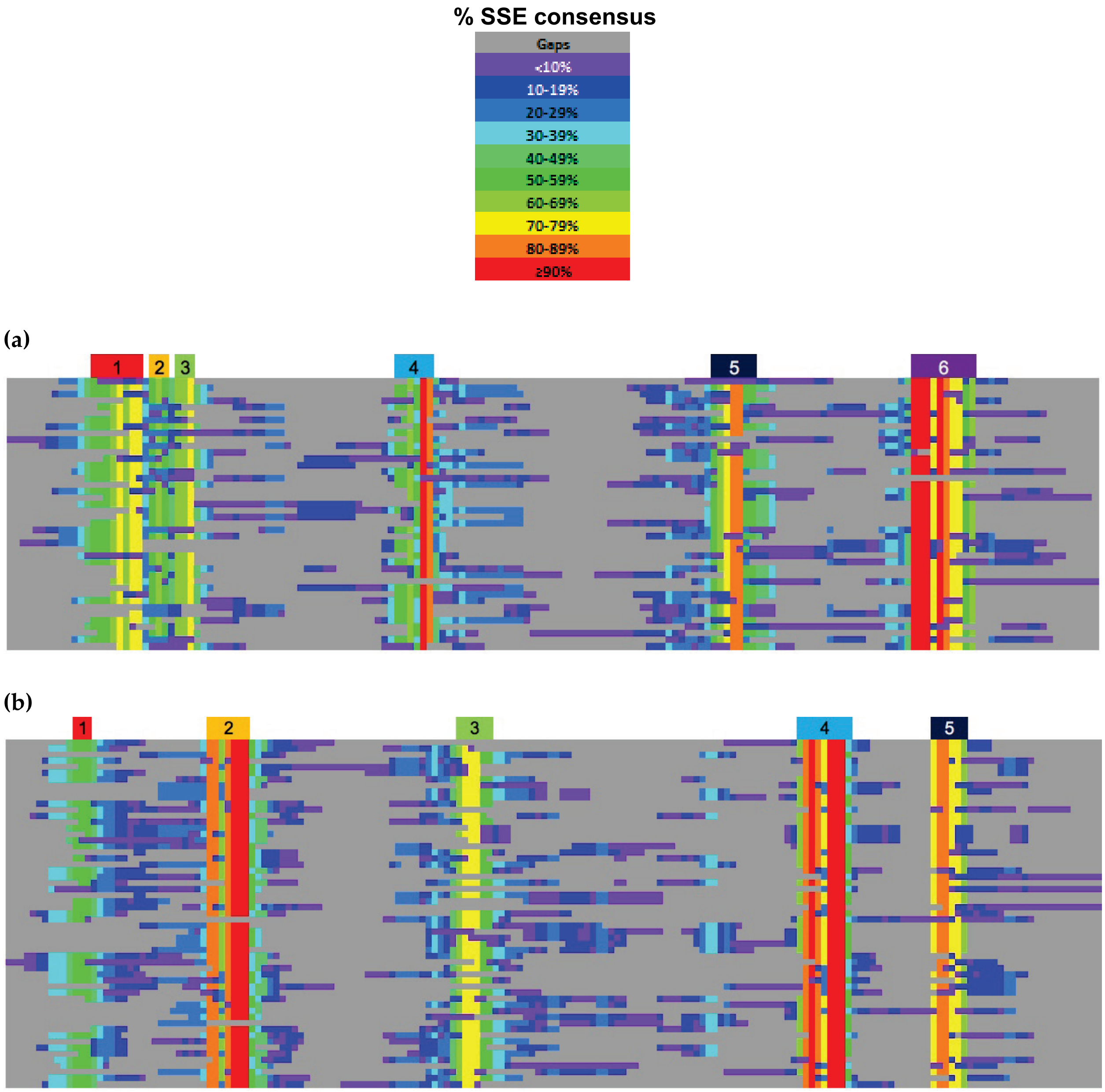

Figure S6. Linear representation of Multiple Secondary Structure Alignment (MSS$A) data from **Figure 4 (a,b)** expanded into a two-dimensional heat map of all aligned secondary structure elements (SSE) for **(a)** dominant-only and **(b)** dominant + suboptimal structures for all 42 AuNR aptamer candidates. Spatial locations of secondary structure consensus domains 1 (red); 2 (orange); 3 (green); 4 (blue); 5 (dark blue); and where relevant 6 (purple) are indicated above each heat map. Each SSE within a consensus domain has a minimum of 50% consensus (i.e. occurs in at least half of the sequences). Color legend (*top*) for the heat map defines the percentage (range) of consensus with all inserted gaps (0% consensus) shown in gray. Due to lateral size restrictions with gap insertions, the identity of each SSE is not provided in any cell.

(a)

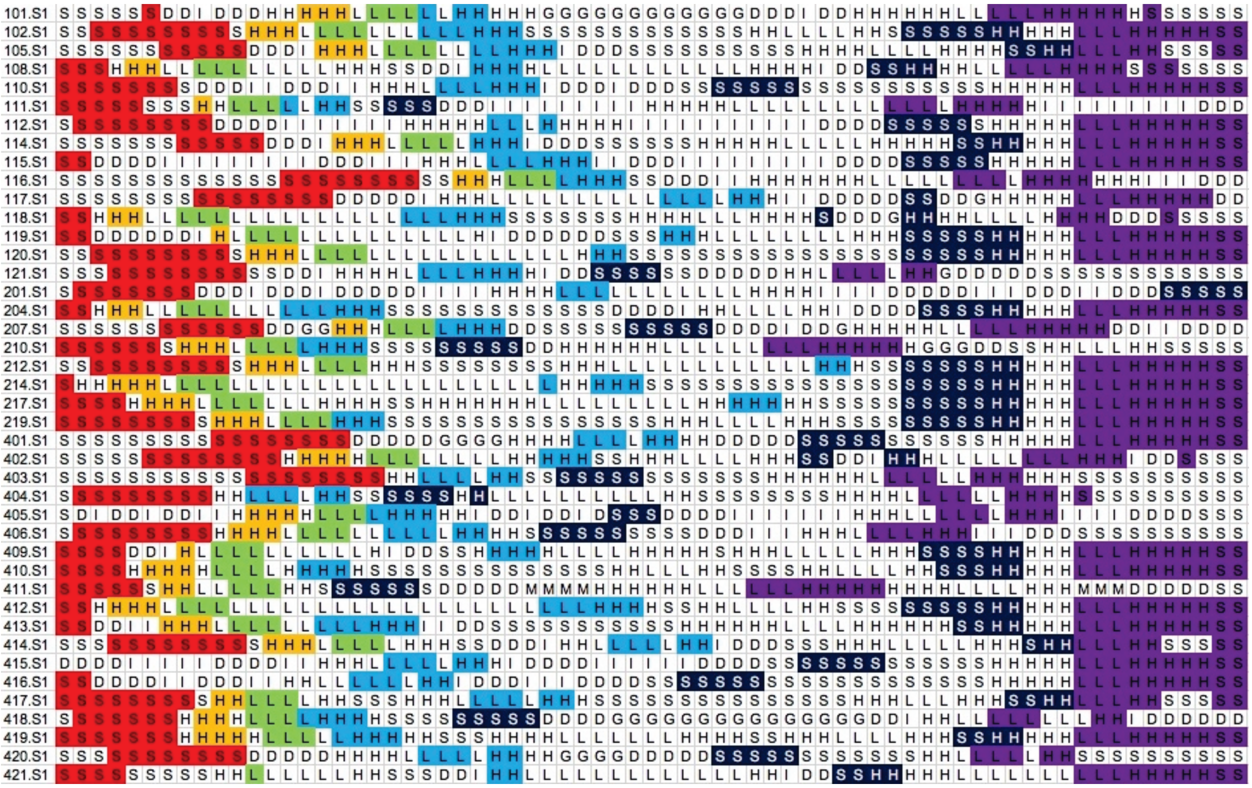

(b)

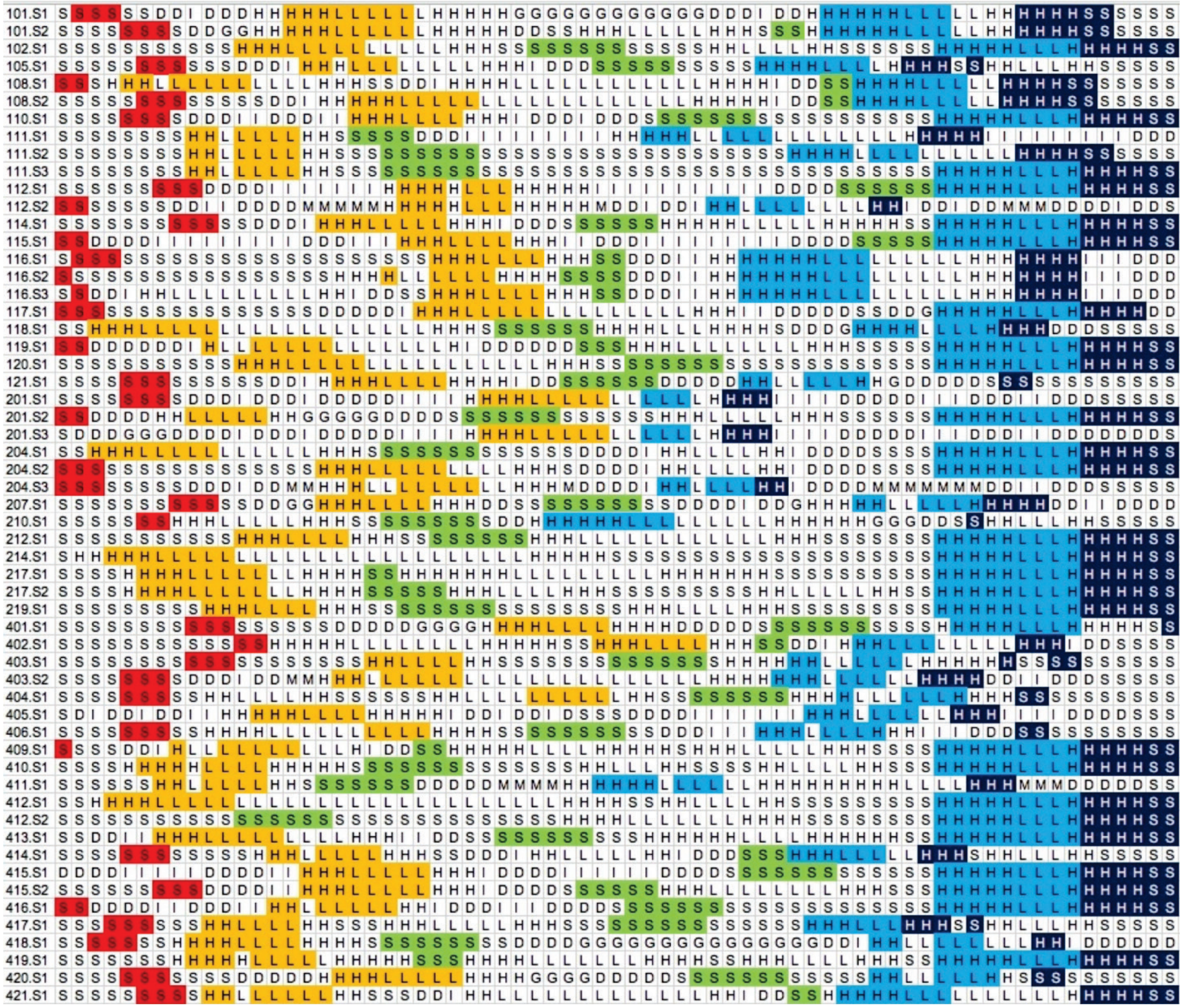

(c)

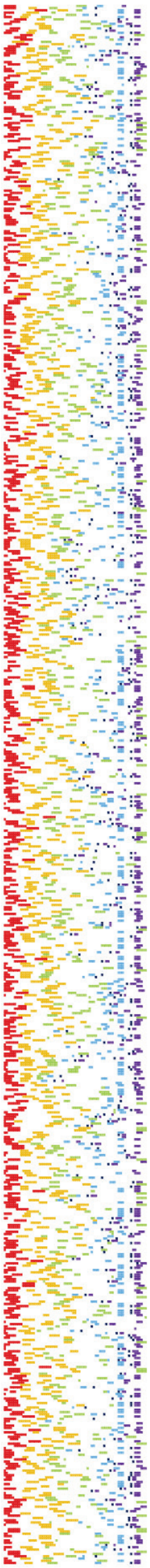

(d)

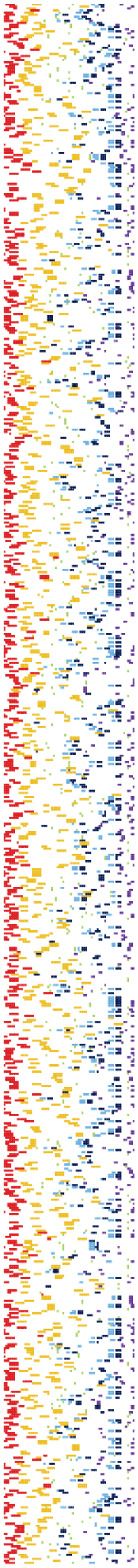

Figure S7. List of all secondary structure strings (SS$) for all 42 AuNR aptamer candidates and 10^3 random sequence populations. All color-coded consensus domains determined from Multiple Secondary Structure Alignment (MSS$A) in **Figure 4** in the main text are re-mapped as a function of numerical base position without gaps for **(a)** dominant-only and **(b)** dominant + suboptimal predicted secondary structures for AuNR aptamers as well as for **(c)** dominant-only and **(d)** dominant + suboptimal predicted secondary structures for random sequence population. Consistent with the color scheme shown in **Figure 4** in the main text, consensus domains here are color-coded as follows: consensus domain 1 (red); consensus domain 2 (yellow); consensus domain 3 (green); consensus domain 4 (light blue); consensus domain 5 (dark blue); and where relevant, consensus domain 6 (purple).

(a)

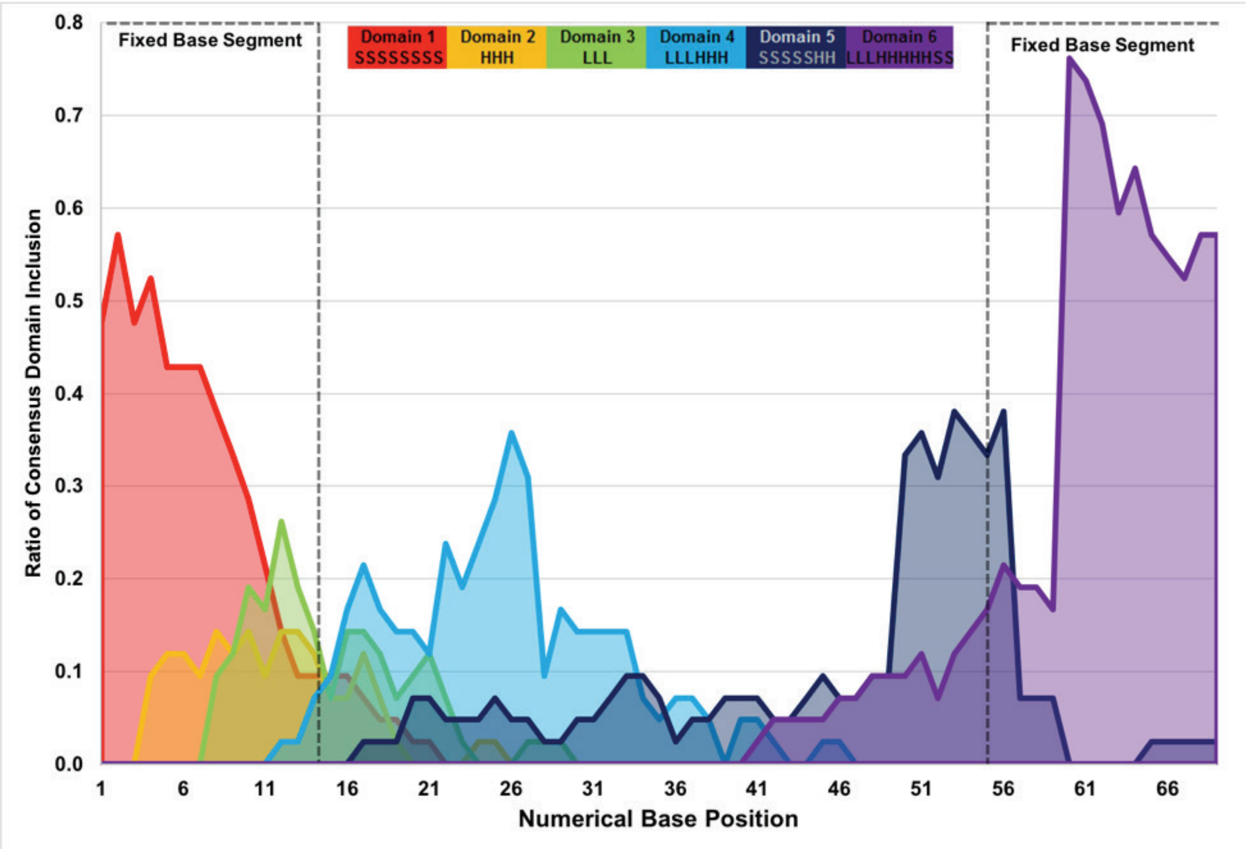
Fixed Base Segment
Domain 1 SSSSSSSS
Domain 2 HHH
Domain 3 LLL
Domain 4 LLLHHH
Domain 5 SSSSSHH
Domain 6 LLLHHHHHSS
Fixed Base Segment
Ratio of Consensus Domain Inclusion
Numerical Base Position

(b)

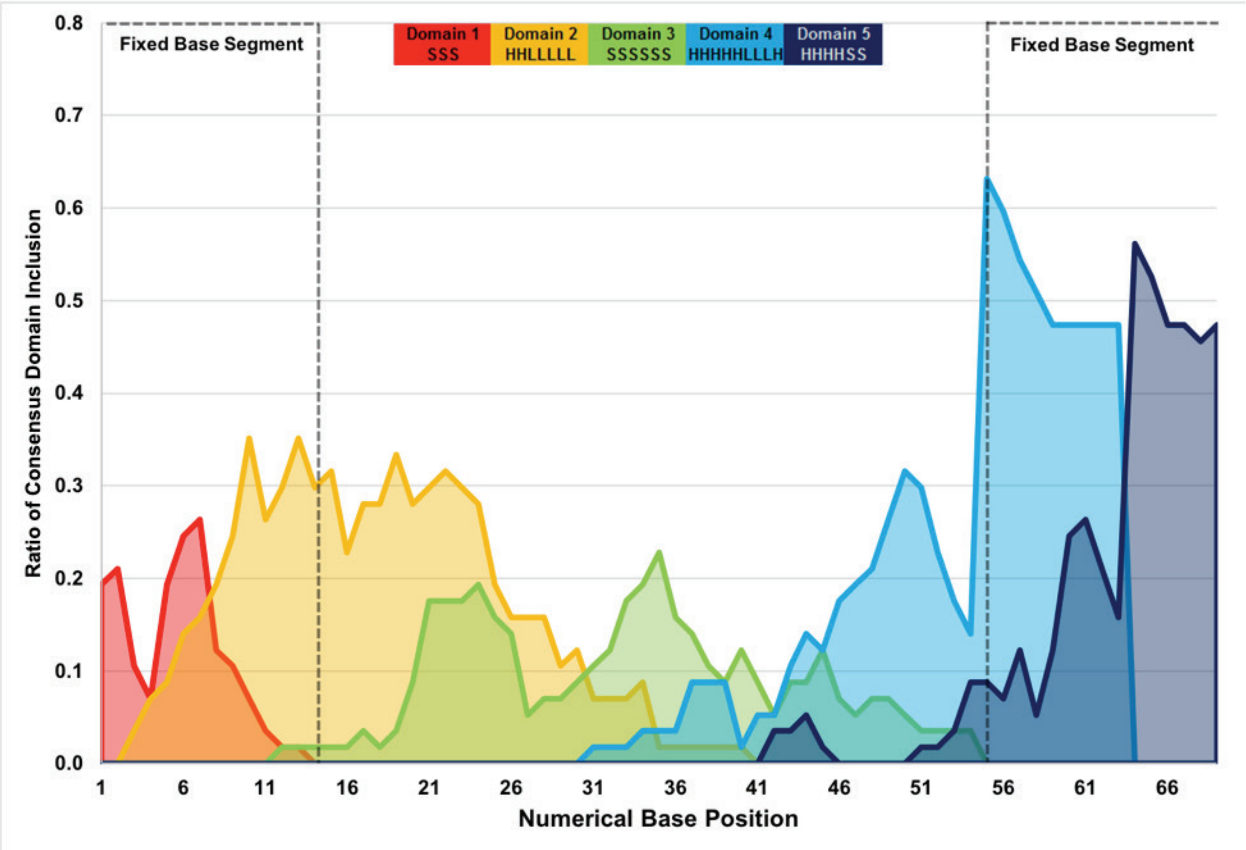
Fixed Base Segment
Domain 1 SSS
Domain 2 HHLLLLL
Domain 3 SSSSSS
Domain 4 HHHHHLLLH
Domain 5 HHHHSS
Fixed Base Segment
Ratio of Consensus Domain Inclusion
Numerical Base Position

(c)

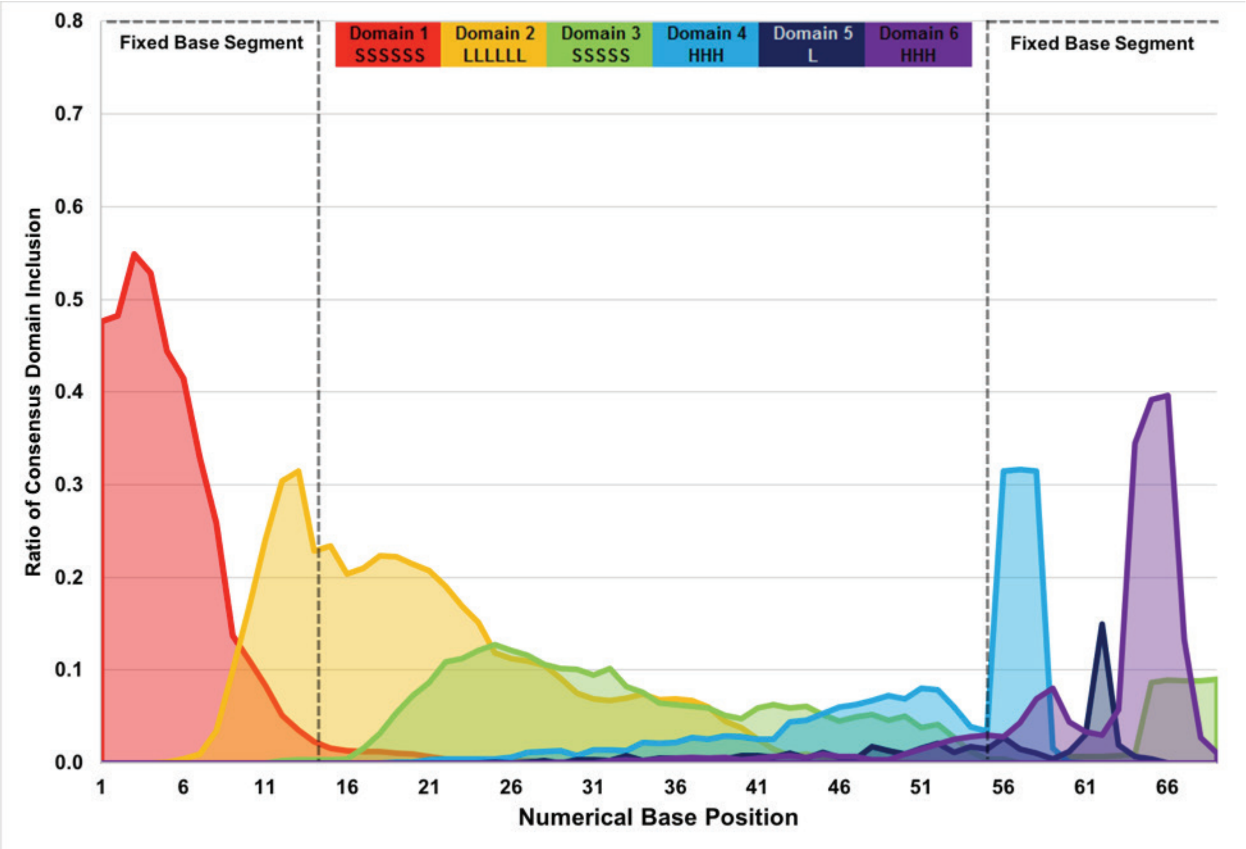
Fixed Base Segment
Domain 1 SSSSSS
Domain 2 LLLLLL
Domain 3 SSSSS
Domain 4 HHH
Domain 5 L
Domain 6 HHH
Fixed Base Segment
Ratio of Consensus Domain Inclusion
Numerical Base Position

(d)

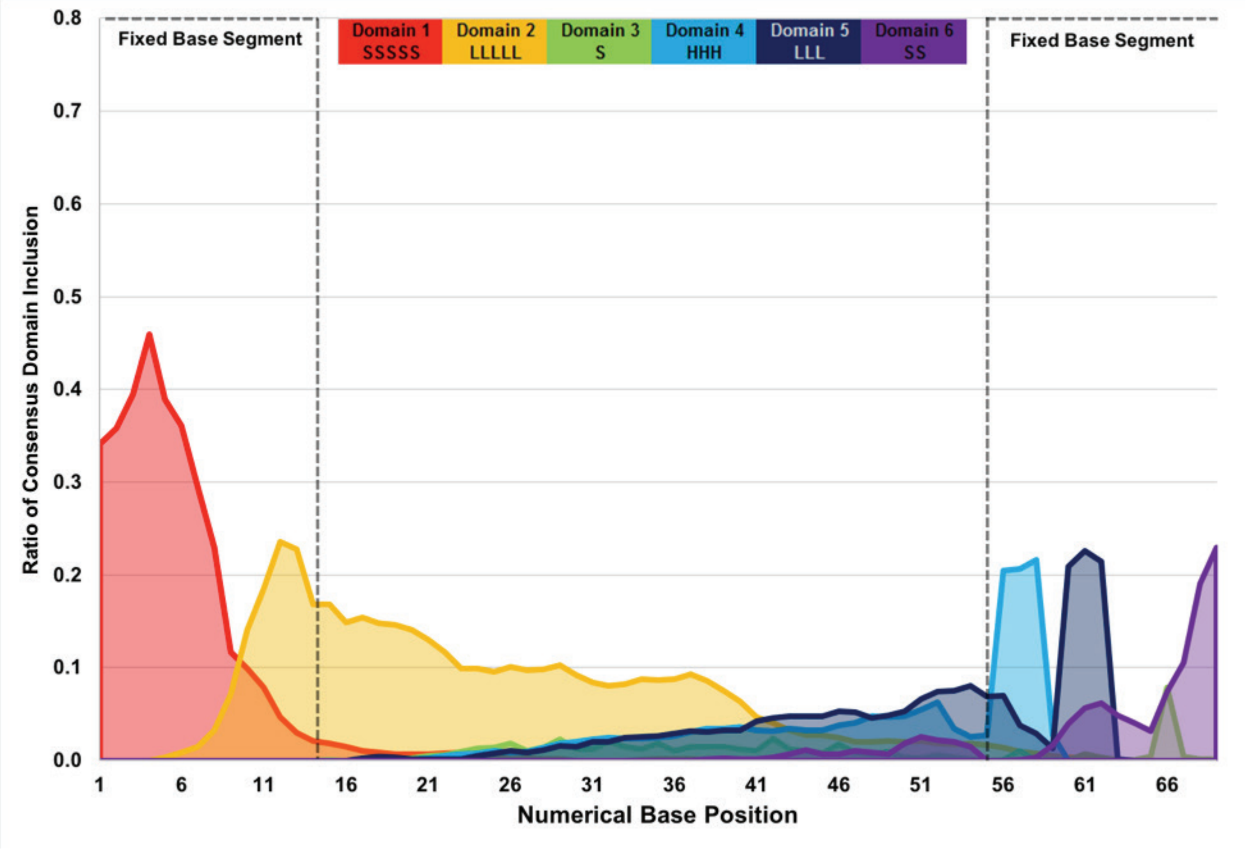
Fixed Base Segment
Domain 1 SSSSS
Domain 2 LLLLL
Domain 3 S
Domain 4 HHH
Domain 5 LLL
Domain 6 SS
Fixed Base Segment
Ratio of Consensus Domain Inclusion
Numerical Base Position

Figure S8. The distribution of secondary structure consensus domains determined from multiple secondary structure string alignment (MSS$A) shown in **Figure 4** is re-mapped here as a function of numerical base position with gaps excluded for **(a)** dominant-only and **(b)** dominant + suboptimal structures for 42 AuNR aptamers as well as for **(c)** dominant-only and **(d)** dominant + suboptimal structures for a random sequence population (10^3 sequences).

Table S3. Numerical information on secondary structure consensus domains for a given sequence set plotted in **Figure S8(a-d)** is listed as follows: associated SS$ of the given domain number; average consensus across the domain (Conserved), statistical frequency of the domain occurrence (Frequency); and fraction of the domain associated with the fixed base segments at either the 5′ or 3′ end (Fraction Fixed Base). Where relevant, standard deviation values are provided alongside the average value.

(a) AuNR aptamer – dominant-only structures

	Domain 1 SSSSSSSS	Domain 2 HHH	Domain 3 LLL	Domain 4 LLLHHH	Domain 5 SSSSSHH	Domain 6 LLLHHHHHSS
**Conserved**	0.661±0.096	0.579±0.036	0.698±0.014	0.683±0.193	0.673±0.146	0.824±0.125
**Frequency**	0.952	0.69	0.714	0.976	0.952	0.976
**Fraction fixed base**	0.923	0.767	0.557	0.029	0.222	0.867

(b) AuNR aptamer - dominant + suboptimal structures

	Domain 1 SSS	Domain 2 HHLLLLL	Domain 3 SSSSSS	Domain 4 HHHHHLLLH	Domain 5 HHHHSS
**Conserved**	0.550±0.037	0.838±0.151	0.649±0.092	0.840±0.165	0.766±0.091
**Frequency**	0.632	0.965	0.807	1.000	1.000
**Fraction fixed base**	1.000	0.372	0.014	0.615	0.935

(c) Random sequence population – dominant-only structures

	Domain 1 SSSSSS	Domain 2 LLLLLL	Domain 3 SSSSS	Domain 4 HHH	Domain 5 L	Domain 6 HHH
**Conserved**	0.670±0.102	0.790±0.146	0.627±0.071	0.688±0.035	0.501±0.000	0.635±0.055
**Frequency**	0.891	0.983	0.813	0.772	0.501	0.735
**Fraction fixed base**	0.976	0.297	0.158	0.483	0.587	0.901

(d) Random sequence population - dominant + suboptimal structures

	Domain 1 SSSSS	Domain 2 LLLLL	Domain 3 S	Domain 4 HHH	Domain 5 LLL	Domain 6 SS
**Conserved**	0.666±0.071	0.853±0.140	0.512±0.000	0.543±0.038	0.670±0.028	0.533±0.030
**Frequency**	0.891	0.983	0.813	0.772	0.501	0.735
**Fraction fixed base**	0.966	0.269	0.222	0.418	0.433	0.842

Table S4. Additional numerical information on secondary structure consensus domains plotted in **Figure S8(a-d)** is listed as follows: average number of position-dependent SSE between secondary structure consensus domains *n* and *n+1* ($d_{n,n+1}$); average number of position-dependent SSE between a secondary structure consensus domain and any previously occurring domain (d_{prior}); average number of position-dependent SSEs between a secondary structure domain and any next occurring secondary structure consensus domain (d_{next}); average number of position-dependent SSEs lost from a central position of a secondary structure consensus domain (d_{loss}). Standard deviation values are reported for each average value.

(a) AuNR aptamer – dominant-only structures

	5′ start	Domain 1	Domain 2	Domain 3	Domain 4	Domain 5	Domain 6
$d_{n,n+1}$	2.25±3.39	2.11±1.89	1.10±0.41	7.52±8.42	15.56±9.78	8.54±7.32	4.22±6.31
d_{prior}	NA	NA	2.41±2.49	1.30±1.15	9.34±8.40	15.25±9.86	9.29±7.97
d_{next}	NA	5.30±6.33	1.10±0.41	7.37±8.31	15.98±9.77	8.30±7.38	NA
d_{loss}	NA	0	0	0	1.07±0.27	2.67±1.53	2.00±1.00

(b) AuNR aptamer - dominant + suboptimal structures

	5′ start	Domain 1	Domain 2	Domain 3	Domain 4	Domain 5
$d_{n,n+1}$	3.25±2.92	11.17±6.07	11.09±7.46	13.59±10.78	2.65±3.98	5.18±6.58
d_{prior}	NA	NA	10.38±6.38	11.13±7.30	15.14±11.82	2.65±3.98
d_{next}	NA	11.22±5.99	13.20±9.99	13.59±10.78	2.65±3.98	NA
d_{loss}	NA	0	1.22±0.43	0	1.53±0.64	1.33±0.58

(c) Random sequence population – dominant-only structures

	5′ start	Domain 1	Domain 2	Domain 3	Domain 4	Domain 5	Domain 6
$d_{n,n+1}$	1.64±2.80	12.02±7.57	13.01±8.65	14.11±10.17	5.18±3.96	1.13±0.41	6.99±7.18
d_{prior}	NA	NA	12.03±7.55	13.12±8.83	15.76±10.87	7.15±7.34	4.36±6.25
d_{next}	NA	12.19±7.85	14.67±9.89	15.17±10.50	5.59±3.50	5.34±9.28	NA
d_{loss}	NA	0	0	0	0	0	0

(d) Random sequence population - dominant + suboptimal structures

	5′ start	Domain 1	Domain 2	Domain 3	Domain 4	Domain 5	Domain 6
$d_{n,n+1}$	2.47±3.74	14.14±10.03	16.54±8.53	9.55±7.03	1.21±0.54	10.07±6.15	5.07±6.90
d_{prior}	NA	NA	14.58±10.49	16.70±8.83	15.06±10.17	5.00±8.79	11.05±7.70
d_{next}	NA	14.33±10.26	18.96±9.71	10.86±7.51	2.01±3.76	12.18±8.17	NA
d_{loss}	NA	0	0	0	0	0	0
